# Emergence of a mutual-growth mechanism in networks evolved by social preference based on indirect utility

**DOI:** 10.1038/s41598-023-48827-6

**Published:** 2023-12-07

**Authors:** Jong-Hyeok Lee, Ken-ichiro Ogawa

**Affiliations:** 1 Department of Computer Science, Tokyo Institute of Technology, Yokohama, Kanagawa 226-8502 Japan; 2 Department of Distribution and Logistics Systems, Ryutsu Keizai University, 3-2-1 Shinmatsudo, Matsudo, 270-8555 Japan

**Keywords:** Complex networks, Applied mathematics, Social evolution, Dynamic networks, Population dynamics, Stochastic networks

## Abstract

Preferential attachment is an important mechanism in the structural evolution of complex networks. However, though resources on a network propagate and have an effect beyond a direct relationship, growth by preferential attachment based on indirectly propagated resources has not been systematically investigated. Here, we propose a mathematical model of an evolving network in which preference is proportional to a utility function reflecting direct utility from directly connected nodes and indirect utility from indirectly connected nodes beyond the directly connected nodes. Our analysis showed that preferential attachment involving indirect utility forms a converged and hierarchical structure, thereby significantly increasing the indirect utility across the entire network. Further, we found that the structures are formed by mutual growth between adjacent nodes, which promotes a scaling exponent of 1.5 between the number of indirect and direct links. Lastly, by examining several real networks, we found evidence of mutual growth, especially in social networks. Our findings demonstrate a growth mechanism emerging in evolving networks with preference for indirect utility, and provide a foundation for systematically investigating the role of preference for indirect utility in the structural and functional evolution of large-scale social networks.

## Introduction

Social preferences contribute to the formation of social networks^1,2^. In particular, preferences proportional to the degree of a node (i.e., the number of links connected to a node), referred to as preferential attachment, simulate the scale-free property of the degree distribution in large-scale evolving networks and serves as a key concept for understanding their formation and the phenomena that occur on them^3–8^. While local mechanisms (e.g., link selection^9^, copying^10,11^, or optimization models^12–14^) sometimes explain the generation of a preference proportional to the degree of a node, a naive human perception of this preference is that of “a person with many friends”.

However, since resources on a network propagate and have an influence beyond direct human relationships, preferences for others are not limited to direct neighbours. In fact, it has been reported that obesity, smoking, and happiness are influenced not only by direct friends but also by friends of friends and even friends of friends of friends^15–17^. According to well-known social theories, weak tie theory and structural hole, which represent the importance of infrequent and non-redundant relationships (i.e. connections to different clusters), suggest that human relationships beyond direct neighbours are important channels for acquiring novel information and that individuals who occupy such structural positions within a network have an advantage in such achievements as employment or promotion^18–20^. In addition, the success of the PageRank algorithm shows that people prefer information on web pages linked by web pages with high importance, rather than those simply linked by a large number of links, which suggests the importance of indirect sources in preferences^21,22^.

Several studies have shown that preferential attachment associated with indirect links beyond direct neighbours links (the sum of the number of the links that neighbours have^23^, the number of links within a second degree of separation^24^, and the betweenness centrality^25^) in an evolving network forms the heavy-tailed degree distribution, the mechanisms of which can complement conventional degree-based preferential attachment through comparison with the structural and temporal characteristics of real networks. Other kinds of models, which are indirectly associated to the preference based on indirect links as links with new nodes are formed through neighbouring nodes, have also been explored^26–29^. However few models are available that investigate theoretically the structural characteristics of networks that social preferences based on indirect links form, and little is known about the social effects of such network structures.

In this study, we investigate the mechanism of structural formation of an evolving network that includes indirect resources as social preferences and its expected social effects. For this purpose, we propose a new mathematical model of evolving networks with a preferential attachment based on utility. Utility is the physical, economic, and psychological effect obtained from various resources, and is usually described as a function consisting of terms of costs and benefits^30,31^. And, from the perspective of network, the utility can be obtained from nodes reachable on the network. We introduce a utility function that considers utility not only from directly connected nodes but also from indirectly connected nodes beyond the directly connected nodes^32–35^. In this utility function, the benefit term of a node that can be obtained from other nodes is designed according to the degree of separation from that node, and, in the case of direct connections, the cost term is designed additionally for the establishment and maintenance of the relationship (see Method for details).

In what follows, we first present our analysis of the temporal networks that a utility-based preferential attachment model evolves under various conditions by adjusting the cost and benefit terms of a utility function. From a macroscopic perspective, our analysis takes into account the structural characteristics of a network and the growth of utility in the entire network; microscopically, we take into account the growth patterns of the direct and indirect links around an individual node through mathematical analysis and numerical simulations. Furthermore, our investigation of growth patterns of direct and indirect links around an individual node in real networks, especially social networks, confirms that the growth approximates the estimation of the preferential attachment model based on indirect utility.

Our results suggest that the utility-based preferential attachment model can provide a foundation for understanding the structural and functional evolution of large-scale social networks. In particular, we show that preferential attachment based on indirect utility stably forms a converged and hierarchical structure. This stability provides robust evidence of emerging mutual growth among adjacent nodes in social networks.

## Results

### Illustrative explanation of the utility-based preferential attachment model

**Figure 1:**
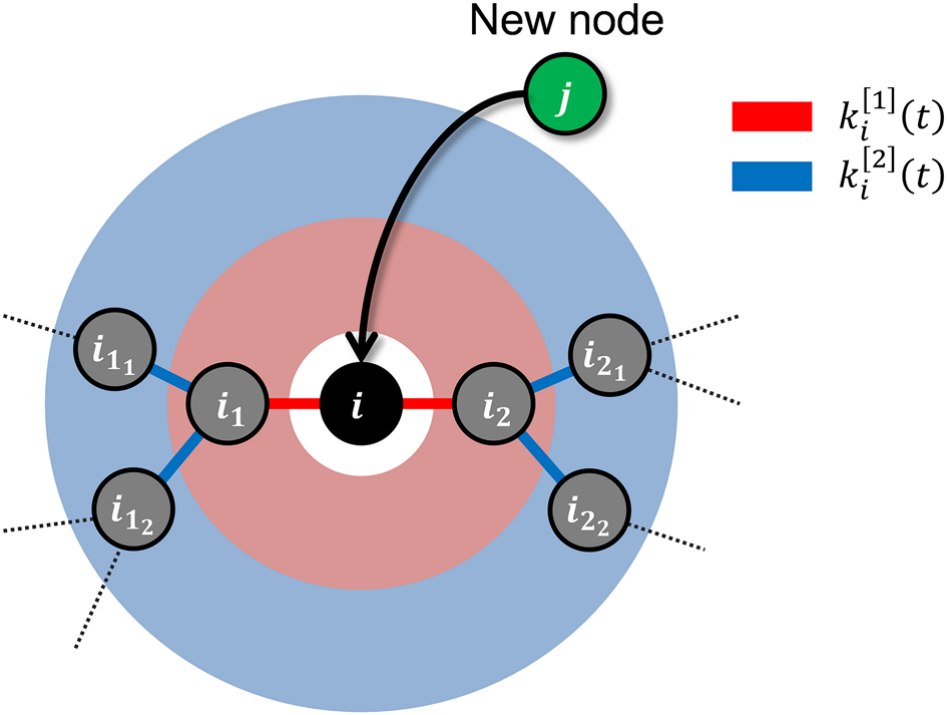
Preferential attachment of the utility-based preferential attachment model. A new node $j$ connects to an existing node $i$ with the preferential attachment probability ${\Pi }_{i}^{[2]}\left({t}_{j}\right)$ at time ${t}_{j}(>{t}_{i})$. ${\Pi }_{i}^{[2]}\left(t\right)$ is proportional to the utility ${u}_{i}^{[2]}\left(t\right)={k}_{i}^{\left[1\right]}\left(t\right)({b}_{1}-c)+{k}_{i}^{\left[2\right]}\left(t\right){b}_{2}$, where ${k}_{i}^{\left[l\right]}\left(t\right) (l=\mathrm{1,2})$ is the number of direct links with the $l$th degree of separation from a node $i$, ${b}_{l}(l=\mathrm{1,2})$ and $c$ are parameters which associate the weight of benefits and costs, respectively (see the “Methods” section). In this example, node $i$ is directly connected to nodes ${i}_{1}$ and ${i}_{2}$, i.e., ${k}_{i}^{\left[1\right]}\left({t}_{j}\right)=2$. Therefore, node $i$ must pay $2c$ as the cost for the direct links to obtain $2{b}_{1}$ from nodes ${i}_{1}$ and ${i}_{2}$ as the direct benefit. Thus, the direct utility is described as $2({b}_{1}-c)$. Also, node $i$ obtains $4{b}_{2}$ as the indirect benefit because it connects indirectly to four nodes, ${i}_{{1}_{1}}$, ${i}_{{1}_{2}}$, ${i}_{{2}_{1}}$, and ${i}_{{2}_{2}}$, i.e., ${k}_{i}^{\left[2\right]}\left({t}_{j}\right)=4$. Thus, the indirect utility becomes $4{b}_{2}$.

Our analysis of the utility-based preferential attachment model takes into consideration the utility within the 2nd degree of separation. The illustration in Fig. 1 serves to explain the preferential attachment of this model. A new node $j$ connects to an existing node $i$ with the preferential attachment probability ${\Pi }_{i}^{[2]}\left({t}_{j}\right)$ at time ${t}_{j}(>{t}_{i})$. ${\Pi }_{i}^{[2]}\left(t\right)$ is proportional to the utility ${u}_{i}^{[2]}\left(t\right)={k}_{i}^{\left[1\right]}\left(t\right)({b}_{1}-c)+{k}_{i}^{\left[2\right]}\left(t\right){b}_{2}$, where ${k}_{i}^{\left[l\right]}\left(t\right) (l=\mathrm{1,2})$ is the number of links at the $l$th degree of separation from node $i$, ${b}_{l}(l=\mathrm{1,2})$ is a nonnegative-valued function representing the benefit of node $i$ obtained from a node within the $l$th degree of separation, $c$ is a positive-valued parameter representing the cost of node $i$ to connect with a node within the $1$st degree of separation. Notably, node $i$ incurs no cost for establishing and maintaining indirect links.

To investigate the effect of indirect utility on the structure and growth of evolving networks, we compared four special cases of the utility-based preferential attachment model, models U_D_, U_I_, U_M+_, U_M-_ with a random-attachment model, U_R_. In every model, a conventional growth rule is used such that a new node joins at each step and makes connections with an existing node with a specific preferential attachment probability. The U_D_ model takes into consideration only the direct utility, i.e., ${\Pi }_{i}^{[2]}\left(t\right)\propto {k}_{i}^{[1]}(t)$. The U_I_ model takes into consideration only the indirect utility, i.e., ${\Pi }_{i}^{[2]}\left(t\right)\propto {k}_{i}^{[2]}(t)$. The U_M+_ and U_M-_ models take into consideration both the direct and indirect utilities. The U_M+_ model represents a case in which the direct benefit exceeds the cost (${b}_{1}=c+1, {b}_{2}=1$), i.e., ${\Pi }_{i}^{[2]}\left(t\right)\propto {k}_{i}^{\left[1\right]}\left(t\right)+{k}_{i}^{\left[2\right]}\left(t\right)$, whereas the U_M-_ model represents a case in which the cost exceeds the direct benefit (${b}_{1}=c-1, {b}_{2}=1$), i.e., ${\Pi }_{i}^{[2]}\left(t\right)\propto {-k}_{i}^{\left[1\right]}\left(t\right)+{k}_{i}^{\left[2\right]}\left(t\right)$. The attachment probability of the U_R_ model is the same for all of the existing nodes in the network.

### The effect of indirect utility on the overall structure and growth of evolving networks

**Figure 2:**
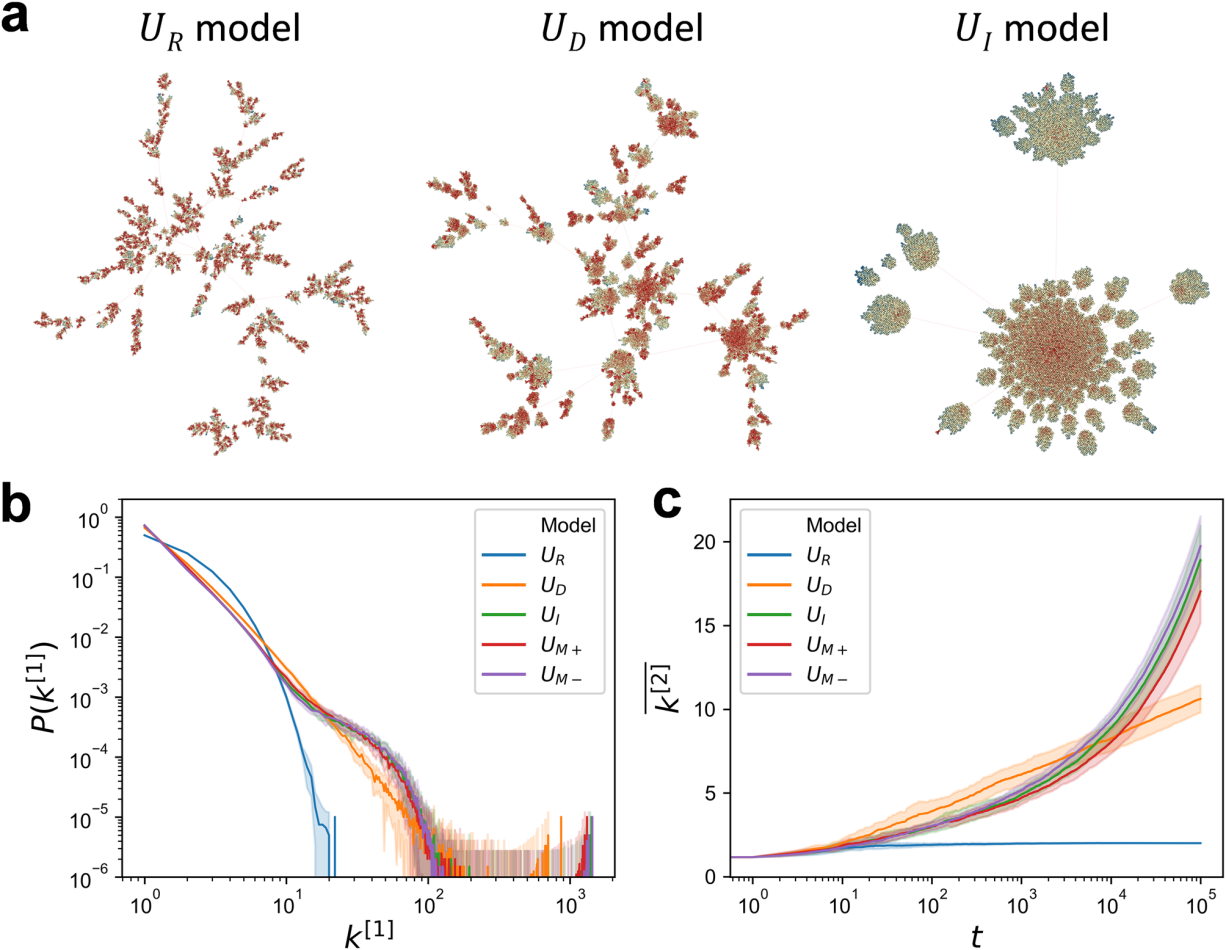
Effect of indirect utility on the structure of the networks formed by the utility-based preferential attachment model. (**a**) An illustration of a network formed by the respective U_R_, U_D_, and U_I_ models at $t=5000$. The red colour represents the local hub, which is a node with ${k}_{i}^{\left[1\right]}$ greater than the surrounding nodes, and it is displayed in blue gradually according to the distance from the nearest local hub. In U_I_ model, a converged and hierarchical structure forms in which many nodes grow sequentially around a very large local hub. (**b**,**c**) The distribution $P(k^{[1]})$ and the growth of the ratio $\overline{{k}^{\left[2\right]}}(t)(\equiv {\sum }_{{{j}}=1}^{{{t}}}{{{k}}}_{{{j}}}^{\left[2\right]}({{t}})/{\sum }_{{{j}}=1}^{{{t}}}{{{k}}}_{{{j}}}^{\left[1\right]}({{t}}))$ in the utility-based preferential attachment models. The solid line represents the average of 20 numerical simulations ($t=100,000$), and the error bar denotes the standard deviation.

Figure 2 shows the effect of indirect utility on the structure of networks formed by the U_R_, U_D_, U_I_, U_M+_, and U_M-_ models. Figure 2a illustrates the networks formed by the U_R_, U_D_, and U_I_ models at around $t=5000$. A node with more direct links than the surrounding nodes, hereafter referred to as a “local hub”, is marked in red, and other nodes are marked in blue as the distance from a local hub increases. The distance is defined as the number of steps taken to find a local hub by recursively searching the neighbour with the most direct links. In the network formed by the U_R_ model, all of the nodes grow evenly, while, in the networks formed by each of the U_D_ and U_I_ models, the growth of a hub with a large number of direct links is conspicuous, resulting in a heavy-tailed degree distribution. In particular, the network formed by the U_I_ model has a converged and hierarchical structure in which many nodes grow sequentially around a very large local hub.

The degree distribution, $P({k}^{[1]})$ of U_R_ (blue line in Fig. 2b) and U_D_ (orange line in Fig. 2b) shows the exponential and the scale-free property, respectively, which is well known as characteristics of the random attachment model and the degree-based preferential attachment model in evolving networks^3^. Meanwhile, the U_I_, U_M+_, and U_M-_ models have a bump property in the degree distribution (green, red, and purple plots). These models commonly have an indirect utility in preference, and it is known that including 2^nd^ degree of separation in preference result in time dependent degree distributions, and gradual collapse of its scaling^24^. As shown in the U_I_ model of Fig. 2a, the bump property of the degree distribution is associated with the converged and hierarchical structure, and it can be seen that it is a common structure formed by indirect utility.

The growth of the indirect links as shown in Fig. 2c also confirms the difference between the models. Here, $\overline{{k }^{\left[2\right]}}\left(t\right)$ is defined as $\overline{{k }^{\left[2\right]}}\left(t\right)\equiv {\sum }_{j=1}^{t}{k}_{j}^{\left[2\right]}\left(t\right)/{\sum }_{j=1}^{t}{k}_{j}^{\left[1\right]}\left(t\right)$. Because ${\sum }_{j=1}^{t}{k}_{j}^{\left[1\right]}\left(t\right)$ can be calculated as ${\sum }_{j=1}^{t}{k}_{j}^{\left[1\right]}\left(t\right)\approx 2t$ in each model, the difference among these models results from ${\sum }_{j=1}^{t}{k}_{j}^{\left[2\right]}\left(t\right)$, which represents indirect benefits among overall network. As Fig. 2c shows, the growth pattern of $\overline{{k }^{\left[2\right]}}\left(t\right)$ for each model is distinct. The U_R_ model shows that the growth pattern of $\overline{{k }^{\left[2\right]}}\left(t\right)$ asymptotically converges to a constant, and the U_D_ model shows that the growth pattern of $\overline{{k }^{\left[2\right]}}\left(t\right)$ is asymptotically proportional to $\mathrm{ln}\sqrt{{\sum }_{j=1}^{t}{k}_{j}^{\left[1\right]}\left(t\right)}$($\approx \mathrm{ln}\sqrt{2t}$) (see Supplementary Information S1 for mathematical analysis). $\mathrm{The growth of }\overline{{k }^{\left[2\right]}}\left(t\right)$ in the U_I_, U_M+_, and U_M-_ models is lower than that in the U_D_ model from the early stage until around $t=\mathrm{5,000}$. However, since $\overline{{k }^{\left[2\right]}}\left(t\right)$ in these models afterwards grows faster, $\overline{{k }^{\left[2\right]}}\left(t\right)$ becomes larger than in the U_D_ model.

The U_M-_, U_I_, and U_M+_ models have the same indirect utility coefficient as 1, but the direct utility coefficients differ as − 1, 0, and 1 (see Eqs. (S29), (S33), and (S37)). Interestingly, the value of $\overline{{k }^{\left[2\right]}}\left(t\right)$ increases in the order of the U_M-_, U_I_, and U_M+_ models as time passes. These differences results from the balance between direct utility and indirect utility in preference. And, it is counterintuitive that higher $\overline{{k }^{\left[2\right]}}$ is observed in the U_M-_ model which penalizes the number of direct links ${k}_{i}^{\left[1\right]}$, since attaching to a node with a high ${k}_{i}^{\left[1\right]}$ is advantageous for the immediate growth of $\overline{{k }^{\left[2\right]}}$, which means $\Delta \overline{{k }^{\left[2\right]}}=2{k}_{i}^{\left[1\right]}$ where $i$ denotes the node attached by a new node. Thus, the structure formed by the preference for ${k}_{i}^{\left[2\right]}$ contributes more to the growth of $\overline{{k }^{\left[2\right]}}$ than the preference for ${k}_{i}^{\left[1\right]}$ as the network grows up.

### The effect of the indirect utility on the growth of the respective nodes in evolving networks

**Figure 3:**
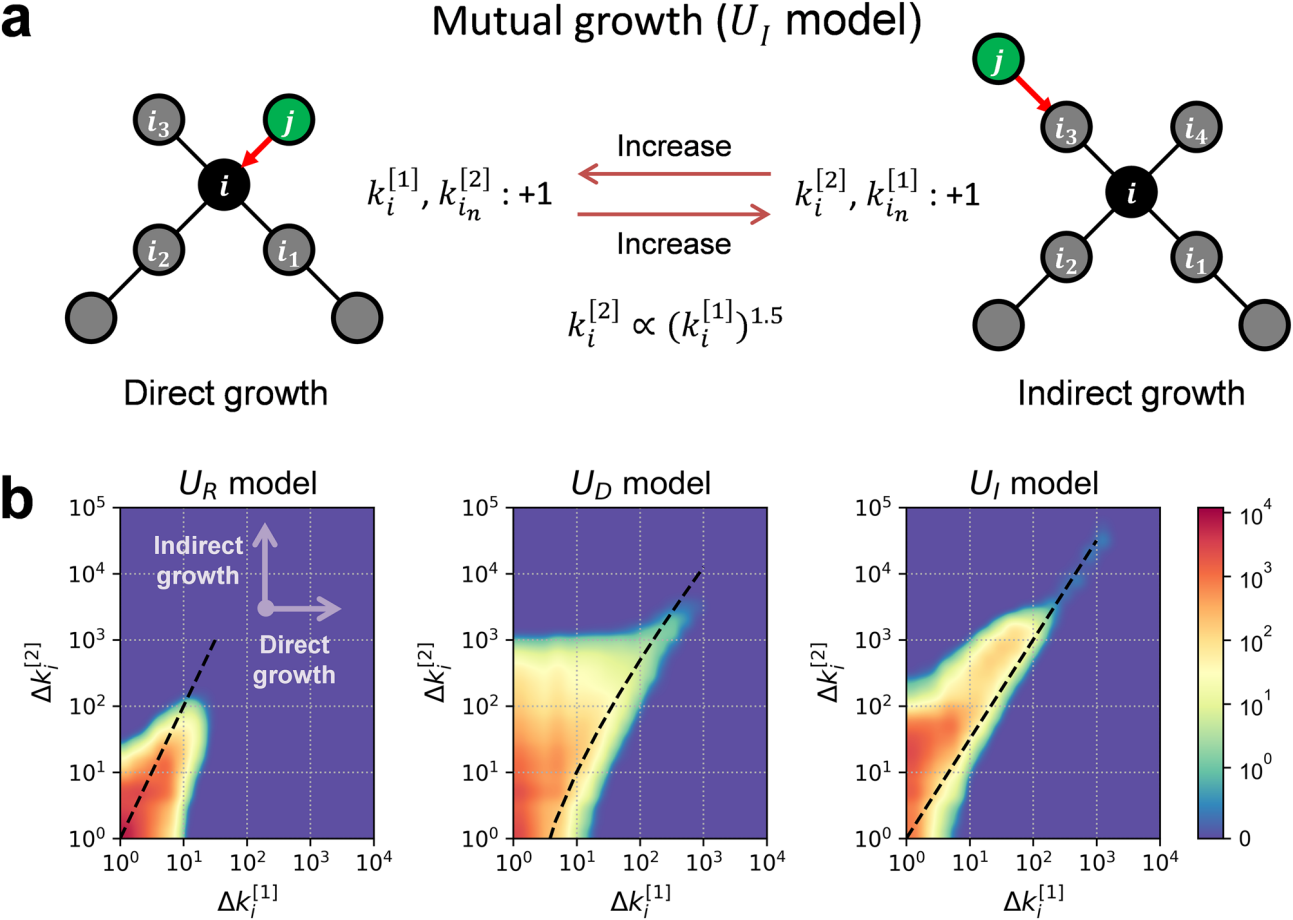
A growth mechanism of the U_I_ model and the relationship between direct and indirect growth in a network formed by the U_R_, U_D_, and U_I_ models. (**a**) A growth mechanism for the networks formed by the U_I_ model. If new node $j$ attached to $i$. When a new node $j$ is attached to node $i$, ${k}_{i}^{\left[1\right]}$ and ${k}_{{i}_{n}}^{\left[2\right]}$ increase, which is reflected in the indirect utility of node ${i}_{n}$, increasing the preference for node ${i}_{n}$. Similarly, When a new node $j$ is attached to node ${i}_{n}$, ${k}_{{i}_{n}}^{\left[1\right]}$ and ${k}_{i}^{\left[2\right]}$ increase, which is reflected in the indirect utility of node $i$, increasing the preference for node $i$. Therefore, a node $i$ and neighbouring nodes ${i}_{n}$ mutually grow through increase each other's preference, and the relationship is established as ${k}_{i}^{\left[2\right]}(t)\propto {({k}_{i}^{\left[1\right]}(t))}^{1.5}$ (see Supplementary Information S2). (**b**) A set of heat maps between $\Delta {k}_{i}^{\left[1\right]}(t)$ and $\Delta {k}_{i}^{\left[2\right]}(t)$, each of which shows the distribution of the direct and indirect growth of nodes in an evolving network simulated numerically by the U_R_, U_D_, and U_I_ models. The horizontal and vertical arrows denote the orientation of the direct and indirect growth. The number of nodes in each area in the heat map is the average value of 20 numerical simulations ($t=\mathrm{100,000}$). The dashed lines are estimates based on mathematical analysis (see Supplementary Information S2). Areas with no growth in direct and indirect growth are excluded for convenience on log scale.

In any case, to understand the rapid growth of $\overline{{k }^{\left[2\right]}}\left(t\right)$ in the U_I_, U_M+_, and U_M-_ models at a late stage, it is necessary to consider the growth mechanism of the network formed by the indirect utility that the three models have in common. Figure 3a shows a possible growth mechanism of the network formed by the U_I_ model, in which the preferential attachment probability is proportional to the indirect utility. As can be seen on the left, when new node $j$ attaches to node $i$ in the existing network, ${k}_{i}^{\left[1\right]}$ increases by one in what is hereafter referred to as “direct growth” from a perspective of node $i$. At the same time, each ${k}_{{i}_{n}}^{\left[2\right]}$ of existing nodes ${i}_{n}$ adjacent to node $i$ increases by one. This increases the preference of nodes ${i}_{n}$, since ${\Pi }_{{i}_{n}}^{[2]}$ is proportional to ${k}_{{i}_{n}}^{\left[2\right]}$ in the U_I_ model. The figure on the right explains the case where a new node $j$ attaches to ${i}_{3}$ which is one of the nodes ${i}_{n}$. This attachment corresponds to “indirect growth” from the point of view of node $i$ and increases ${k}_{{i}_{3}}^{\left[1\right]}$ and ${k}_{i}^{\left[2\right]}$ by one, and increases the preference of node $i$. In this way, a specific node $i$ and adjacent nodes ${i}_{n}$ grow mutually by alternating direct growth and indirect growth. Such growth is hereafter referred to as a “mutual growth”.

Figure 3b shows the patterns of direct growth and indirect growth of each node in an evolving network numerically simulated by the U_R_, U_D_, and U_I_ models, respectively. Direct growth and indirect growth are set to $\Delta {k}_{i}^{\left[1\right]}(t)\equiv {k}_{i}^{\left[1\right]}(t)-{{k}_{i}^{\left[1\right]}}_{int}$ and $\Delta {k}_{i}^{\left[2\right]}(t)\equiv {k}_{i}^{\left[2\right]}(t)-{{k}_{i}^{\left[2\right]}}_{int}$ to eliminate the initial value dependency that occurs when node $i$ first enters the network, and the colour of the heat map indicates the number of nodes in that region. As Fig. 3b indicates, the U_I_ model shows the most positive correlation between ${k}_{i}^{\left[1\right]}(t)$ and ${k}_{i}^{\left[2\right]}(t)$ in these models. In the U_R_ model direct and indirect growth of nodes are relatively insignificant, and In the U_D_ model, high indirect growth is observed in nodes with high direct growth, but no clear correlation across nodes as a whole can be seen. Our mathematical analysis in Supplementary Information S2 demonstrates that the scale of ${k}_{i}^{\left[2\right]}(t)$ of nodes grown considerably in the U_R_, U_D_, and U_I_ models is approximately ${({k}_{i}^{\left[1\right]}(t))}^{2}$, ${k}_{i}^{\left[1\right]}(t)\mathrm{log}{k}_{i}^{\left[1\right]}(t)$, and ${({k}_{i}^{\left[1\right]}\left(t\right))}^{1.5}$, respectively.

In particular, in the U_I_ model, two patterns of growth mechanism appear according to the direction of mutual growth (see Supplementary Information S2 for details). A node in which mutual growth of ${k}_{i}^{\left[1\right]}\left(t\right)$ and ${k}_{i}^{\left[2\right]}\left(t\right)$ dominates the growth mechanism leads the growth of outer areas (${k}_{i}^{\left[l\right]}\left(t\right)$ ($l>2$)) and becomes the centre of growth. This growth is hereafter referred to as “active growth”. However, if the influence by the outer area ${k}_{i}^{\left[3\right]}\left(t\right)$ is dominant in the growth mechanism, mutual growth occurs in the opposite direction to the above, where ${k}_{i}^{\left[3\right]}\left(t\right)$ leads to the growth of ${k}_{i}^{\left[2\right]}\left(t\right)$ and then ${k}_{i}^{\left[2\right]}\left(t\right)$ leads to the growth of ${k}_{i}^{\left[1\right]}\left(t\right)$. This growth is hereafter referred to as “passive growth”. Therefore, excluding external influences ${k}_{i}^{\left[l\right]}\left(t\right)$ ($l>2$), that is, considering only the growth of ${k}_{i}^{\left[1\right]}\left(t\right)$ and ${k}_{i}^{\left[2\right]}\left(t\right)$ of node $i$, the indirect preferential attachment leads a scaling exponent of 1.5 (i.e. ${k}_{i}^{\left[2\right]}\left(t\right)\sim {({k}_{i}^{\left[1\right]}\left(t\right))}^{1.5}$). Thus, mutual growth by the indirect preferential attachment is ideally expected to have a scaling exponent of 1.5, but a scaling exponent greater than 1.5 appear in the case of passive growth, which is attracted to external growth (the U_I_ model in Fig. 3b). These distinct growth patterns, active growth and passive growth explain the nodes that are the centre of growth and the nodes that propagate the growth in the network structure, respectively, and explain how the local mechanism of mutual growth between direct growth and indirect growth (Fig. 3a) leads to the emergence of a converged and hierarchical structure that the growth of nodes propagate outward from the centre (U_I_ model in Fig. 2a).

### The effect of indirect utility on the structure and growth of nodes in real social networks

The mathematical analysis and numerical simulations just described indicate that the mutual growth between direct growth and indirect growth with a scaling exponent of 1.5 in the networks formed by the preferential attachment based on indirect utility. However, observing the mutual growth in real networks and interpreting it as growth by indirect utilities can be challenging, since even from a perspective of the utility-based preferential attachment model, the benefit and cost coefficients can vary depending on the environment in which the network is formed, and also there can be nodes with various preferences in evolving networks. In this regard, our mathematical analysis indicates that in the utility-based preferential attachment model with a preference for indirect utility (e.g., U_M+_ and U_M-_ models), a node with active growth forms the scaling exponent of 1.5, regardless of the benefit and cost coefficients (see Supplementary Information S3). Further, we confirm the dominant effect of indirect utility in a network grown by sequentially attaching nodes with preferential attachments of U_R_, U_D_, and U_I_ models, and also confirm the scaling exponent of 1.5 in sufficiently grown nodes (see Supplementary Information S4). These robust results support the assumption that there is a preference based on indirect utility in the evolving mechanism of the network when the scale of mutual growth in sufficiently grown nodes is close to 1.5, from the point of view of the utility-based preferential attachment model.

To confirm the existence of a preference based on indirect utility in social networks, we investigated the scaling exponent of the indirect growth ${\Delta k}_{i}^{\left[2\right]}\left(t\right)$ of each node with respect to the direct growth ${\Delta k}_{i}^{\left[1\right]}\left(t\right)$ in two real temporal social networks, YouTube and Facebook, which was well observed over time^36,37^. The data on the YouTube network records the friendships that grew for 165 days, and the data on the Facebook network records the comment activities on wall pages of New Orleans users for 850 days.

**Figure 4:**
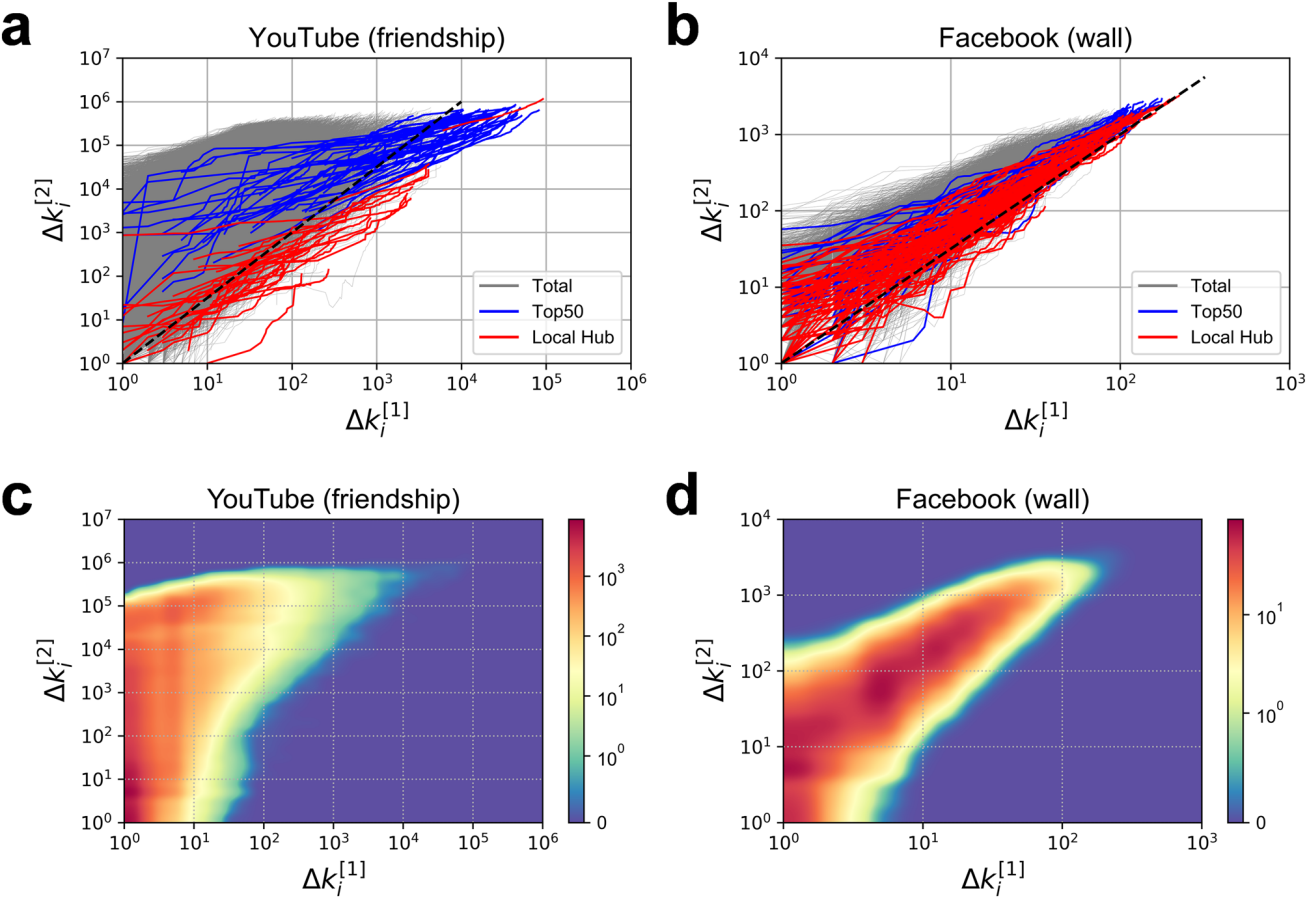
Relationship between the direct and indirect growth of nodes in real temporal social networks. (**a**,**b**) The results of tracking the direct growth, $\Delta {k}_{i}^{\left[1\right]}\left(t\right)$, and indirect growth, ${\Delta k}_{i}^{\left[2\right]}\left(t\right)$, of each node in the YouTube and Facebook networks. Local hubs (red) are nodes with ${k}_{i}^{\left[1\right]}$ larger than the neighbouring nodes in the final state, and Top50 (blue) corresponds to 50 nodes in the order of higher ${k}_{i}^{\left[1\right]}$ in the final state. The dashed line represents the relationship ${k}_{i}^{\left[2\right]}(t)\propto {({k}_{i}^{\left[1\right]}(t))}^{1.5}$ which is the theoretical estimate of the mutual growth mechanism (see Supplementary Information S2). (**c**,**d**) Heat maps in the relationship between $\Delta {k}_{i}^{\left[1\right]}(t)$ and $\Delta {k}_{i}^{\left[2\right]}(t)$ which shows the distribution of the direct and indirect growth of nodes in the YouTube and Facebook networks in the final state (same with Fig. 3b).

Figure 4a,b show the results of tracking $\Delta {k}_{i}^{\left[1\right]}\left(t\right)$ and ${\Delta k}_{i}^{\left[2\right]}\left(t\right)$ of each node in the YouTube and Facebook networks, respectively. As confirmed in the numerical simulation and mathematical analysis, the 1.5 scale due to mutual growth is evident in the sufficiently grown nodes of active growth. Therefore, we coloured the top 50 nodes (blue) in order of high $\Delta {k}_{i}^{\left[1\right]}$, and local hubs (red) with high $\Delta {k}_{i}^{\left[1\right]}$ compared to neighbouring nodes that are likely to be in an active growth state. The dashed lines represent the growth of the nodes with scaling exponents of 1.5 in the relationship between $\Delta {k}_{i}^{\left[1\right]}\left(t\right)$ and ${\Delta k}_{i}^{\left[2\right]}\left(t\right)$. In the YouTube network, the scaling exponent of the local hubs has a value lower than 1.5 while, in the Facebook network, the value is close to 1.5. The top 50 nodes in each social network show a trend similar to that of the scaling exponent for the local hubs. This result indicates the possibility that some indirect utility relates deeply to the major mechanism of the growth of the nodes in the Facebook network whereas other mechanisms may explain the growth of nodes in the YouTube network.

Figure 4c,d present heat maps in the relationship between $\Delta {k}_{i}^{\left[1\right]}$ and $\Delta {k}_{i}^{\left[2\right]}$ of each node entering the YouTube and Facebook networks, respectively. Figure 4d clearly shows that the indirect links in the Facebook network grow with a scaling exponent of 1.5, a result similar to that of the U_I_ model (Fig. 3b). However, no sign of such growth is observable in the YouTube network shown in Fig. 4c. This result suggests that there are mutual growths between the direct and indirect growths in the Facebook network.

Consideration of the meaning of the links in each network is necessary to interpret these results. The friendship links observed on the YouTube network require the mutual consent of each user. However, since this relationship is not publicly revealed to others, its indirect utility may be difficult to observe. In the Facebook network, by contrast, the comments on a user’s wall are publicly exposed to others. Therefore, posting a comment on the wall naturally increases a user’s exposure to others and the probability that they will visit and comment on the wall. The formation of links triggered by this indirect exposure can be seen as preferential attachment based on indirect utility and suggests the possibility that the direct and indirect growth of nodes affect each other mutually.

Next, we consider the scaling exponent of $\Delta {k}_{i}^{\left[2\right]}$ of each node with respect to $\Delta {k}_{i}^{\left[1\right]}$ in real static social networks using various categories of datasets. Unlike temporal networks, static networks cannot directly observe the scaling exponent of $\Delta {k}_{i}^{\left[2\right]}$ of each node with respect to $\Delta {k}_{i}^{\left[1\right]}$ over time, but we roughly approximate the growth scale using the fact that, as Figs. 3b and 4 show, the influence of the scale exponent becomes dominant in the local hubs. Specifically, $\Delta {k}_{i}^{\left[2\right]}\propto {(\Delta {k}_{i}^{\left[1\right]})}^{\alpha }$ is set estimated roughly as ${k}_{i}^{\left[2\right]}={({k}_{i}^{\left[1\right]})}^{\alpha }$ for the local hubs satisfying ${k}_{i}^{\left[1\right]}>50$. From the Stanford large network dataset collection, we select 34 undirected and unweighted networks, the categories of which are classified as autonomous systems (3 networks), collaboration networks (5 networks), social networks (23 networks), and Wikipedia (3 networks)^38^. The selection criteria targeted categories grouped into the same type, and only cases where the category contained three or more undirected and unweighted network samples were selected (see “Methods” for details).

**Figure 5:**
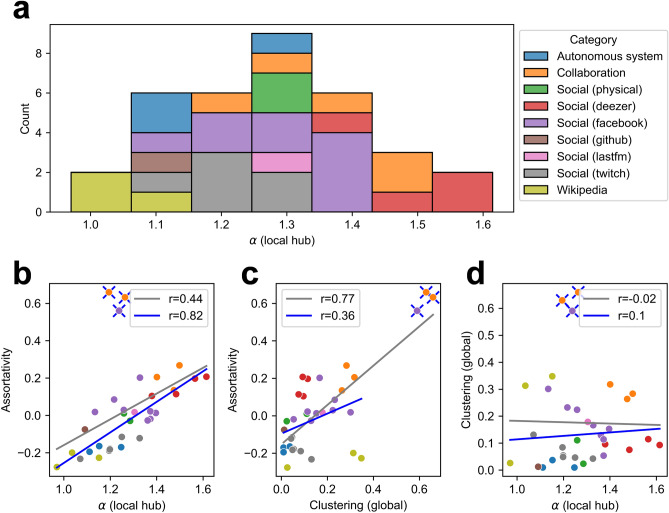
Averaged scaling exponent of the indirect growth of local hubs with respect to the direct growth and the relationship among the scaling exponent, cluster coefficient, and degree assortativity coefficient in real static networks. (**a**) The average scaling exponent $\alpha$ calculated from the local hubs in each of 34 undirected and unweighted networks for which the categories are classified as autonomous systems (3 networks), collaboration networks (5 networks), social networks (23 networks), and Wikipedia (3 networks). (**b–d**) The correlation between scaling exponent $\alpha$ and the degree assortativity coefficient $r$, between the cluster coefficient $C$ and $r$, and between $\alpha$ and $C$. The grey line represents the correlation of the entire network, and the blue line represents the correlation except for networks with exceptionally large values of $r$ and $C$. The value in the legend is a *Pearson*’s correlation coefficient. The colour of the dot is the same as the categories identified in (**a**).

Figure 5a shows the averaged value of $\alpha$ calculated from the local hubs of each of the various networks. The social networks in social media (*deezer*, *facebook*, physical location-based online social media, *Last.fm*), and collaboration networks between researchers show a large value of $\alpha$ while the networks of Wikipedia, autonomous systems, and some social networks (*github*, *twitch*) show a small value of $\alpha$. We are unable to completely explain the complex growth mechanism of these real networks, but the preferential attachment based on indirect utility provides a partial explanation for values of $\alpha$ ranging from 1 to 1.5. In the networks in which social interactions take place, as described above, local hubs have a value of $\alpha$ close to 1.5, indicating that the local hubs in the social networks grow not alone but together with the surrounding nodes and suggesting that preferential attachment based on indirect utility may have contributed to this growth.

Further, we investigated the relationship between the scaling exponent $\alpha$ and two representative indicators of the network structure, the “assortativity coefficient” and “clustering coefficient”. The assortativity coefficient $r$ indicates a correlation between the degrees of a node and the neighbouring nodes across the network^39^. The clustering coefficient $C$ indicates the average of the ratio of closed triplets among the possible triplets for each node in the whole network. Previous studies indicate that there is a correlation between $r$ and $C$^40,41^. As Fig. 5c shows, there is a strong correlation between $r$ and $C$, with a *Pearson*’s correlation coefficient of 0.77. However, after exclusion of the three networks with exceptionally large values of $r$, $C$ decreases to 0.36. By contrast, as Fig. 5b clearly shows, with the exclusion of the three networks, the *Pearson*’s correlation coefficient between $r$ and $\alpha$ increases from 0.44 to 0.82. The three networks taking very large values of $r$ and $C$ suggest that the clustering of the nodes around the hubs is reflected in the large values of $r$. Meanwhile, as Fig. 5d shows, there is no significant relationship between $\alpha$ and $C$.

It is well known that the relationship between $r$ and $C$ appears as a positive correlation in social networks^39^. And, it has been suggested that community structure and clustering can have a positive effect on degree assortativity^40–42^. However, the results reported above indicate that the effect of $\alpha$ can explain the positivity of $r$ in a different, even better direction from the effect of $C$. Accordingly, the mutual growth pattern by the preferential attachment based on indirect utility may be an important growth mechanism of the positive degree assortativity in social networks.

## Discussion

Preferential attachment has become an important mechanism for understanding the evolution of social networks^3–8^. However, compared to the interest in the mechanism by which such a preference can occur in a physical sense^9–14^, there has been a lack of interest in a framework that deals with preferences in a cognitive sense, especially regarding indirect utility^15–22^. In this study, we have introduced the utility-based preferential attachment model by focusing on social preference in human cognition, investigating in particular the effect of the preference attachment based on indirect utility on the structure and growth of evolving networks. Our numerical simulations and mathematical analysis demonstrated that, in evolving networks, converged and hierarchical structures where the growths of nodes propagate outward from the centre are stably emerged through the preferential attachment based on indirect utility. In addition, we show that this growth mechanism promotes the mutual growth of direct growth and indirect growth with a scaling exponent of 1.5 from a perspective of each node, and observe the mutual growth patterns in real networks, especially social networks.

The main finding of our numerical simulations and mathematical analyses is the converged and hierarchical structures (see Fig. 2a) formed by the influx of nodes that prefer nodes with high indirect utility. This structure is a robust outcome confirmed not only in a model taking into consideration only indirect utility (the U_I_ model), but also in models taking into consideration both the direct and indirect utilities (e.g., the U_M+_ and U_M-_ models$,$ see Supplementary Information S3), and a model in which nodes with different preferences are mixed (see Supplementary Information S4). Our work reveals how preferential attachment based on indirect utility forms a microscopic mutual growth mechanism (see Fig. 3a), and how this mechanism differentiates into active growth and passive growth, and leads to the emergence of the converged and hierarchical structure. Therefore, in a society where there is a continuous influx of people who prefer indirect utility, this structure can emerge deterministically.

In the utility-based preferential attachment model, utility serves not only as to preferences for the attachment to nodes in the network, but also as the utility that each node can obtain from the network. In an evolving network, the total indirect utility increases in proportion to the number of direct links of the node attached by a new node. Thus, the preference proportional to the number of direct links explicitly means the preference for increasing indirect utility. However, despite the absence of an explicit relationship to increasing indirect utility, the preferential attachment based on indirect utility rapidly increases the indirect utility of the whole network, exceeding the total indirect utility of the network formed by the preferential attachment based on direct utility over time (see Fig. 2c). The rapid growth is an outcome not due to a preference of nodes for indirect utility per se, but due to the mechanism of mutual growth between adjacent nodes in the network based on the preference and the converged and hierarchical structure that emerged from it. This increasing pattern of utility is likely the underlying mechanism by which people increase utility in large-scale evolving networks such as cities^43,44^, especially considering that the number of direct connections of people can be finite under cognitive constraints^45,46^.

In the conventional preferential attachment models, the growth of a node depended on the elapsed time after entering the network^3^ or on a fitness of individual nodes^7^. Therefore, it is common not to associate preference with the growth of a node, and preference has been understood as an intrinsic property of each node or as physical mechanisms^9–14^. However, in the preferential attachment based on indirect utility, attachment by preference and outcome by attachment are dependent mutually. For example, a node attached to a location near centre area of a converged and hierarchical structure grows faster than nodes that do not. And, if the outcome of attachment by a preference leads to greater growth, a feedback loop that reinforces the preference can form. The analysis in a network model grown by sequentially attaching nodes with preferential attachments of the U_R_, U_D_, and U_I_ models also supports that nodes with a preference for indirect utility are more advantageous for the growth of direct link and indirect link than nodes with no preference or nodes with preference for direct utility (see Supplementary Information S4). Therefore, preferences for indirect utility are not just an intrinsic property, but a property that can be stably formed by preferential attachment and feedback by its outcomes. It is meaningful to consider this dependent growth and the possibility of forming indirect preferences as its background in social networks. There is dependent growth in the formation of networks between people. For example, popularity among peers in adolescence has a contagious effect^47,48^, and research on weak tie theory and structural holes shows that links that expand indirect connections could be a source of growth^18–20^. Under these environmental conditions, people do not simply choose nodes that are random or have a high probability of being connected. People adaptively and strategically evaluate the utility of the nodes with which to form connections, and also pursue their own growth through the choice. Our model shows that this dependent growth mechanism can emerge simply from a preferential attachment based on indirect utility.

The strong correlation between the scaling exponent of indirect growth and degree assortativity (Fig. 5b) opens up new possibilities for positive degree assortativity appeared in evolving social networks^40^. The conventional interpretations of the origin of degree assortativity have been focused on the clustering effect^40–42,49^, since the more closed the open-triad is, the more clusters with similar degree are formed. However, the degree of a node adjacent to node $i$ includes not only the links of clusters formed with other nodes adjacent to node $i$, but also the links formed on outside of the nodes adjacent to node $i$. The scaling exponent of indirect growth of a node can be described as an indicator independent from the clustering coefficient in that it excludes the link between the adjacent nodes of node $i$. The fact that this indicator correlates strongly with the degree assortativity indicates that the mutual growth mechanism arising from the preferential attachment based on indirect utility may contributes to the positivity of the degree assortativity, which is an important characteristic of social networks.

One of the major limitations of the utility-based preferential attachment model is that preferential attachment requires computing the utility information of all nodes in the network. The assumption that a new node can explore preferences for all nodes is unrealistic, and conventional preferential attachment models have supported feasibility through models based on local mechanisms^9–14^. For the preferential attachment based on indirect utility, a local mechanism can be devised in which a link is formed after moving randomly to a node with a shortest distance of 2 from a point selected randomly so that effects of indirect utility can be obtained. In this case, the mutual growth mechanism around a node, which is a local growth mechanism, would work identically with the utility-based preferential attachment model, but the size or formation dynamics of the macroscopic structure may be different. For example, our numerical simulations form converged and hierarchical structures around very few dominant active growth nodes, but if a local growth mechanism is applied, the structures centred on more nodes with active growth can be formed throughout the network. Also, preferences for indirect utility can be considered even in connections between existing nodes for realistic extensions. A recent study shows that selection to increase betweenness centrality forms an ultra-small world network through a game theoretical framework^50^. Preferences for indirect utility are likely to make a similar contribution, as it could be a practical way to increase betweenness centrality. With these extensions, future studies can construct more realistic models. In addition, the definition of the local hub (i.e., a node with higher degree than neighbouring nodes) introduced to estimate the active growth state needs to be refined. Future studies can attempt to increase the observation accuracy of mutual growth through some strategies such as removing nodes with a larger degree among neighbouring nodes, or nodes showing unusual growth patterns. Moreover, the growth mechanism may not always be stable throughout the entire process of network formation. For example, though it is rough, in the case of the YouTube friendship network (see Fig. 4c), the growth pattern of the nodes with prominent growth (Top50) appears to change into a growth pattern with different scaling exponent around ${k}^{\left[1\right]}\approx 100$. In this way, the growth mechanism of real networks may vary depending on the growth scale of nodes, and future studies can consider these heterogeneous growth patterns. Lastly, in this study, we defined the most basic form of utility function to be linearly proportional to the number of directly connected nodes and indirectly connected nodes. However, the model may need to be adjusted depending on the context of the network under investigation. For example, if it is a type of network where the number of human relationships is limited by the cognitive limits of the nodes, such as Dunbar's number^45,46^, a non-linear function in which utility is saturated as the number of nodes increases can be considered. Depending on the nature of the utility or the perception of the utility, there may be cases where the benefit or cost is fixed and not proportional to the number of links^51^. Future research could attempt to investigate the variation and robustness of network structural evolution and mutual growth scaling by adjusting the model in different contexts.

In summary, our study showed growth mechanisms and structural features that emerge from the influx of nodes that prefer indirect utility. These results contribute to the understanding of evolving mechanisms of social networks, and the methodologies can be widely applied to the investigation of microscopic and macroscopic growth patterns of evolving networks.

## Methods

### Utility-based preferential attachment model

We propose as a mathematical model of evolving networks the utility-based preferential attachment model

1 $$\frac{d}{dt}{k}_{i}^{\left[1\right]}\left(t\right)=m{\Pi }_{i}^{[n]}\left(t\right),$$

where

2 $${\Pi }_{i}^{[n]}\left(t\right)=\frac{{u}_{i}^{\left[n\right]}\left(t\right)}{{\sum }_{j=1}^{t}{u}_{j}^{\left[n\right]}\left(t\right)},$$

3 $${u}_{i}^{[n]}\left(t\right)={\sum }_{l=1}^{n}{k}_{i}^{\left[l\right]}\left(t\right){b}_{i(l)}-{k}_{i}^{\left[1\right]}\left(t\right){c}_{i}.$$

Here, ${\Pi }_{i}^{[n]}\left(t\right)$ is the preferential attachment probability normalized as

4 $${0\le\Pi }_{i}^{[n]}\left(t\right)\le 1 \quad \mathrm{for}\,\, \forall t\left(\ge {t}_{i}\right).$$

${u}_{i}^{[n]}\left(t\right)$ is the utility function of node $i$, which was attached to a node on the existing network at time ${t}_{i}$, at time $t (\ge {t}_{i})$. ${u}_{i}^{[n]}\left(t\right)$ represents the utility that node $i$ obtains from all of the nodes within the *n*^th^ degree of separation on the network. The first term on the right side of this function,

5 $${b}_{i}^{[n]}\left(t\right)\equiv {\sum }_{l=1}^{n}{k}_{i}^{\left[l\right]}\left(t\right){b}_{i(l)},$$

denotes the benefit of node $i$ obtained from all of the nodes within the *n*th degree of separation from, and the second term,

6 $${c}_{i}^{\left[1\right]}(t)\equiv {k}_{i}^{\left[1\right]}\left(t\right){c}_{i},$$

denotes the cost that node $i$ needs to pay to directly connect to the nodes with the 1st degree of separation. Here, ${c}_{i}$ is a parameter with a positive value representing the cost that node $i$ needs to pay to connect to a node with the 1^st^ degree of separation from node $i$. ${k}_{i}^{\left[l\right]}\left(t\right)$ is the number of links within the *l*th degree of separation from node $i$, which was attached to a node on the existing network at time ${t}_{i}$, at time $t (\ge {t}_{i})$. ${b}_{i(l)}$ denotes a function with a positive value representing the benefit of node $i$ obtained from a node with the *l*th degree of separation. Also, $m$ is the number of links attaching a new node to existing nodes.

${u}_{i}^{[n]}\left(t\right)$ is also rewritten as

7 $${u}_{i}^{[n]}\left(t\right)={u}_{i}^{\left[n\right]\left[D\right]}\left(t\right)+{u}_{i}^{\left[n\right]\left[I\right]}\left(t\right),$$

where

8 $${u}_{i}^{\left[n\right]\left[D\right]}\left(t\right)\equiv {k}_{i}^{\left[1\right]}\left(t\right)\left({b}_{i(1)}-{c}_{i}\right),$$

9 $${u}_{i}^{\left[n\right]\left[I\right]}\left(t\right)\equiv {\sum }_{l=2}^{n}{k}_{i}^{\left[l\right]}\left(t\right){b}_{i(l)}.$$

Here, ${u}_{i}^{\left[n\right]\left[D\right]}\left(t\right)$ is the direct utility term, that is, the utility of node $i$ obtained from all of the nodes with the 1st degree of separation, and ${u}_{i}^{\left[n\right]\left[I\right]}\left(t\right)$ is the indirect utility term, that is, the utility of node $i$ obtained from all of the nodes within the range between the 2nd and *n*th degrees of separation.

### The utility-based preferential attachment models with ${\varvec{n}}=2,$ including the U_D_, U_I_, U_M+_, and U_M-_ models

In what follows, we set ${c}_{i}$ for arbitrary node $i$ to the same value $c$ and ${b}_{i(l)}$ for arbitrary node $i$ to the same function ${b}_{(l)}$ for simplicity. Notably, ${b}_{(l)}$ is written as simply ${b}_{l}$. We restrict the distance between nodes here to $n=2$. Thus, the utility-based preferential attachment model is reduced to

10 $$\frac{d}{dt}{k}_{i}^{\left[1\right]}\left(t\right)=m{\Pi }_{i}^{[2]}\left(t\right),$$

where

11 $${\Pi }_{i}^{[2]}\left(t\right)=\frac{{u}_{i}^{\left[2\right]}\left(t\right)}{{\sum }_{j=1}^{t}{u}_{j}^{\left[2\right]}\left(t\right)},$$

12 $$\begin{aligned} u_{i}^{\left[ 2 \right]} \left( t \right) &= u_{i}^{\left[ 2 \right]\left[ D \right]} \left( t \right) + u_{i}^{\left[ 2 \right]\left[ I \right]} \left( t \right) \nonumber \\ &\quad = g\left( {c,b_{1} } \right)k_{i}^{\left[ 1 \right]} \left( t \right) + b_{2} k_{i}^{\left[ 2 \right]} \left( t \right), \end{aligned}$$

13 $$g\left(c,{b}_{1}\right)\equiv {b}_{1}-c.$$

Then, ${\Pi }_{i}^{[2]}\left(t\right)$ is described as

14 $${\Pi }_{i}^{[2]}\left(t\right)=\frac{g(c,{b}_{1}){k}_{i}^{\left[1\right]}\left(t\right)+{b}_{2}{k}_{i}^{\left[2\right]}\left(t\right)}{{\sum }_{j=1}^{t}\left\{g(c,{b}_{1}){k}_{j}^{\left[1\right]}\left(t\right)+{b}_{2}{k}_{j}^{\left[2\right]}\left(t\right)\right\}},$$

and Eqs. (11) and (12) show that the utility-based preferential attachment model with $n=2$ is described as

15 $$\frac{d}{dt}{k}_{i}^{\left[1\right]}\left(t\right)=m\frac{g\left(c,{b}_{1}\right){k}_{i}^{\left[1\right]}\left(t\right)+{b}_{2}{k}_{i}^{\left[2\right]}\left(t\right)}{{\sum }_{j=1}^{t}\left\{g\left(c,{b}_{1}\right){k}_{j}^{\left[1\right]}\left(t\right)+{b}_{2}{k}_{j}^{\left[2\right]}\left(t\right)\right\}},$$

16 $$\frac{d}{dt}{k}_{i}^{\left[2\right]}\left(t\right)=m\frac{{b}_{2}{\left({k}_{i}^{\left[1\right]}\left(t\right)\right)}^{2}+\left\{g\left(c,{b}_{1}\right)-{b}_{2}\right\}{k}_{i}^{\left[1\right]}(t)+g\left(c,{b}_{1}\right){k}_{i}^{\left[2\right]}\left(t\right)+{b}_{2}{k}_{i}^{[3]}(t)}{{\sum }_{j=1}^{t}\left\{g\left(c,{b}_{1}\right){k}_{j}^{\left[1\right]}\left(t\right)+{b}_{2}{k}_{j}^{\left[2\right]}\left(t\right)\right\}}$$

(see Supplementary Information S3). This model is hereafter referred to as the “utility-based preferential attachment model” for simplicity. In this study, we analyse the case of $m=1$.

The U_D_ model represents the situation satisfying ${u}_{i}^{\left[2\right]}\left(t\right)={u}_{i}^{\left[2\right]\left[D\right]}\left(t\right)$, which is realized in the condition ${b}_{2}=0$, and the substitution of this condition into Eqs. (13) and (14) shows that ${\Pi }_{i}^{\left[2\right]}\left(t\right)$ becomes

17 $${\Pi }_{i}^{\left[2\right]}\left(t\right)=\frac{{k}_{i}^{\left[1\right]}\left(t\right)}{{\sum }_{j=1}^{t}{k}_{j}^{\left[1\right]}\left(t\right)}.$$

This is the same preferential attachment probability as the BA model, and this means that considering only direct utility in the preference, the utility-based preferential attachment model is reduced to the BA model. Then, the substitution of ${b}_{2}=0$ into Eqs. (15) and (16) shows that the U_D_ model is mathematically described as

18 $$\frac{d}{dt}{k}_{i}^{\left[1\right]}\left(t\right)=\frac{{k}_{i}^{\left[1\right]}\left(t\right)}{{\sum }_{j=1}^{t}{k}_{j}^{\left[1\right]}\left(t\right)},$$

19 $$\frac{d}{dt}{k}_{i}^{\left[2\right]}\left(t\right)=\frac{{{k}_{i}^{\left[1\right]}\left(t\right)+k}_{i}^{\left[2\right]}\left(t\right)}{{\sum }_{j=1}^{t}{k}_{j}^{\left[1\right]}\left(t\right)}.$$

The U_I_ model represents the situation satisfying ${u}_{i}^{\left[2\right]}\left(t\right)={u}_{i}^{\left[2\right]\left[I\right]}\left(t\right)$, which is realized in the condition ${b}_{1}=c$, and substituting this condition into Eqs. (13) and (14) indicates that ${\Pi }_{i}^{\left[2\right]}\left(t\right)$ becomes

20 $${\Pi }_{i}^{\left[2\right]}\left(t\right)=\frac{{k}_{i}^{\left[2\right]}\left(t\right)}{{\sum }_{j=1}^{t}{k}_{j}^{\left[2\right]}\left(t\right)}.$$

Then, the substitution of ${b}_{1}=c$ into Eqs. (15) and (16) shows that the U_I_ model is mathematically described as

21 $$\frac{d}{dt}{k}_{i}^{\left[1\right]}\left(t\right)=\frac{{k}_{i}^{\left[2\right]}\left(t\right)}{{\sum }_{j=1}^{t}{k}_{j}^{\left[2\right]}\left(t\right)},$$

22 $$\frac{d}{dt}{k}_{i}^{\left[2\right]}\left(t\right)=\frac{{\left({k}_{i}^{\left[1\right]}\left(t\right)\right)}^{2}-{k}_{i}^{\left[1\right]}\left(t\right)+{k}_{i}^{\left[3\right]}(t)}{{\sum }_{j=1}^{t}{k}_{j}^{\left[2\right]}(t)}.$$

The U_M+_ model represents the situation satisfying ${u}_{i}^{\left[2\right]}\left(t\right)={u}_{i}^{\left[2\right]\left[D\right]}\left(t\right)+{u}_{i}^{\left[2\right]\left[I\right]}\left(t\right)={k}_{i}^{\left[1\right]}\left(t\right)+{k}_{i}^{\left[2\right]}\left(t\right)$, which is realized in the condition ${b}_{1}=c+1, {b}_{2}=1$, and the substitution of this condition into Eqs. (13) and (14) shows that ${\Pi }_{i}^{\left[2\right]}\left(t\right)$ becomes

23 $${\Pi }_{i}^{\left[2\right]}\left(t\right)=\frac{{k}_{i}^{\left[1\right]}\left(t\right)+{k}_{i}^{\left[2\right]}\left(t\right)}{{\sum }_{j=1}^{t}\left({k}_{i}^{\left[1\right]}\left(t\right)+{k}_{j}^{\left[2\right]}\left(t\right)\right)}.$$

This is the same preferential attachment probability as the K2 model. Then, the substitution of ${b}_{1}=c+1, {b}_{2}=1$ into Eqs. (15) and (16) shows that the U_M+_ model is mathematically described as

24 $$\frac{d}{dt}{k}_{i}^{\left[1\right]}\left(t\right)=\frac{{k}_{i}^{\left[1\right]}\left(t\right)+{k}_{i}^{\left[2\right]}\left(t\right)}{{\sum }_{j=1}^{t}\left({k}_{i}^{\left[1\right]}\left(t\right)+{k}_{j}^{\left[2\right]}\left(t\right)\right)},$$

25 $$\frac{d}{dt}{k}_{i}^{\left[2\right]}\left(t\right)=\frac{{\left({k}_{i}^{\left[1\right]}\left(t\right)\right)}^{2}+{k}_{i}^{\left[2\right]}(t)+{k}_{i}^{\left[3\right]}(t)}{{\sum }_{j=1}^{t}\left({k}_{i}^{\left[1\right]}\left(t\right)+{k}_{j}^{\left[2\right]}\left(t\right)\right)}.$$

The U_M-_ model represents the situation satisfying ${u}_{i}^{\left[2\right]}\left(t\right)={u}_{i}^{\left[2\right]\left[D\right]}\left(t\right)+{u}_{i}^{\left[2\right]\left[I\right]}\left(t\right)=-{k}_{i}^{\left[1\right]}\left(t\right)+{k}_{i}^{\left[2\right]}\left(t\right)$, which is realized in the condition ${b}_{1}=c-1, {b}_{2}=1$, and the substitution of this condition into Eqs. (13) and (14) shows that ${\Pi }_{i}^{\left[2\right]}\left(t\right)$ becomes

26 $${\Pi }_{i}^{\left[2\right]}\left(t\right)=\frac{-{k}_{i}^{\left[1\right]}\left(t\right)+{k}_{i}^{\left[2\right]}\left(t\right)}{{\sum }_{j=1}^{t}\left(-{k}_{i}^{\left[1\right]}\left(t\right)+{k}_{j}^{\left[2\right]}\left(t\right)\right)}.$$

Then, the substitution of ${b}_{1}=c-1, {b}_{2}=1$ into Eqs. (15) and (16) shows that the U_M-_ model is mathematically described as

27 $$\frac{d}{dt}{k}_{i}^{\left[1\right]}\left(t\right)=\frac{-{k}_{i}^{\left[1\right]}\left(t\right)+{k}_{i}^{\left[2\right]}\left(t\right)}{{\sum }_{j=1}^{t}\left(-{k}_{i}^{\left[1\right]}\left(t\right)+{k}_{j}^{\left[2\right]}\left(t\right)\right)},$$

28 $$\frac{d}{dt}{k}_{i}^{\left[2\right]}\left(t\right)=\frac{{\left({k}_{i}^{\left[1\right]}\left(t\right)\right)}^{2}-2{k}_{i}^{\left[1\right]}\left(t\right)-{k}_{i}^{\left[2\right]}(t)+{k}_{i}^{\left[3\right]}(t)}{{\sum }_{j=1}^{t}\left(-{k}_{i}^{\left[1\right]}\left(t\right)+{k}_{j}^{\left[2\right]}\left(t\right)\right)}.$$

### The criteria for selecting a static social network in Stanford large network dataset collection

The Stanford large network dataset collection^38^ divides network datasets into 23 categories. Our selection criteria are as follows: (1) a category in which the properties of nodes can be grouped into the same type, (2) containing at least three network samples to discuss general properties of the category, (3) an unweighted and undirected network like our mathematical model, and (4) a network in which the local hub's degree has grown sufficiently as in the analysis criteria (${k}_{i}^{\left[1\right]}>50$). The categories that met our criteria are “autonomous systems”, “social networks”, “location-based online social networks”, “collaboration networks”, and “Wikipedia”, of which “social networks” and “location-based online social networks” are classified into “social networks” types together. Two networks are excluded as exceptions. “*ego-Facebook*” network in the “social networks” category is not of interest because it is a dataset about the ego network, and “*as-Skitter*” network in “autonomous systems” is excluded because it is too large to analyse. As a result, 34 networks met our criteria: autonomous systems (3 networks), collaboration networks (5 networks), social networks (23 networks), and Wikipedia (3 networks).

### Supplementary Information

Supplementary Information.

## Data Availability

The datasets analysed during this study are publicly available at WOSN 2008, WOSN 2009 Data Sets^36,37^ from Max Planck Institute for Software Systems, https://socialnetworks.mpi-sws.org/data-wosn2008.html, https://socialnetworks.mpi-sws.org/data-wosn2009.html, and Stanford Large Network Dataset Collection^38^, https://snap.stanford.edu/data/.

## Code availability

Source code related to the utility-based preferential attachment model is available at https://github.com/mons1220/utility-model.
