# Supplementary material for: Emergence of a mutual-growth mechanism in networks evolved by social preference based on indirect utility
Source: Sci Rep. 2023 Dec 7;13:21680. doi: 10.1038/s41598-023-48827-6 (PMC10709339; doi:10.1038/s41598-023-48827-6)
Supplement: Supplementary file 1 — Supplementary Information. [file 41598_2023_48827_MOESM1_ESM.docx]

**Supplementary Information**

**Emergence of Mutual-Growth Mechanisms in Networks Evolved by Social Preference Based on Indirect Utility**

**S1: Calculation of** $\bar{\boldsymbol{\Delta k}^{\boldsymbol{[2]}}}\boldsymbol{(t)}$

When $m=1$, by the following equation [24],

$$\sum_{j=1}^{t} \left( k_{j}^{\left[ 1 \right]}\left( t \right)+k_{j}^{\left[ 2 \right]}\left( t \right) \right)\left( \equiv k^{\left[ 1 \right]}\left( t \right)+k^{\left[ 2 \right]}\left( t \right) \right)=\sum_{j=1}^{t} \left( k_{j}^{\left[ 1 \right]}\left( t \right) \right)^{2}$$

$$\to\sum_{j=1}^{t} k_{j}^{\left[ 1 \right]}\left( t \right)+\sum_{j=1}^{t} k_{j}^{\left[ 2 \right]}\left( t \right)=\sum_{j=1}^{t} \left( k_{j}^{\left[ 1 \right]}\left( t \right) \right)^{2}$$

$$\to2t+\sum_{j=1}^{t} k_{j}^{\left[ 2 \right]}\left( t \right)=\sum_{j=1}^{t} \left( k_{j}^{\left[ 1 \right]}\left( t \right) \right)^{2}$$

$$\to\sum_{j=1}^{t} k_{j}^{\left[ 2 \right]}\left( t \right)=\sum_{j=1}^{t} \left( k_{j}^{\left[ 1 \right]}\left( t \right) \right)^{2}-2t,\cdots\left( S1 \right)$$

$\bar{k^{[2]}}(t)$ for $t\gg t_{i}$ is rewritten as

$$\bar{k^{\left[ 2 \right]}}\left( t \right)=\frac{\sum_{j=1}^{t} k_{j}^{\left[ 2 \right]}\left( t \right)}{\sum_{j=1}^{t} k_{j}^{\left[ 1 \right]}\left( t \right)}$$

$$=\frac{\sum_{j=1}^{t} \left( k_{j}^{\left[ 1 \right]}(t) \right)^{2}-2t}{2t}$$

$$=\frac{\sum_{j=1}^{t} \left( k_{j}^{\left[ 1 \right]}(t) \right)^{2}}{2t}-1.\cdots(S2)$$

In the U_R_ model, since $k_{i}^{\left[ 1 \right]}(t)$ temporally develops in accordance with

$$\frac{d}{dt}k_{i}^{\left[ 1 \right]}\left( t \right)=\frac{1}{t+m_{int}-1},\cdots(S3)$$

the solution is obtained as

$$k_{i}^{\left[ 1 \right]}\left( t \right)=m\left( \mathrm{In} \frac{t+m_{int}-1}{t_{i}+m_{int}-1}+1 \right)\cdots(S4)$$

under the initial condition,

$$k_{i}^{\left[ 1 \right]}\left( t_{i} \right)=m.\cdots(S5)$$

Here, $m_{int}$ is the number of nodes at the initial time of network growth. For $m=1$ and $t\gg t_{i}$, Eq. (S4) can be approximated as

$$k_{i}^{\left[ 1 \right]}\left( t \right)=\mathrm{In} \frac{t}{t_{j}}+1.\cdots(S6)$$

Then, substituting Eq. (S6) into Eq. (S2), we obtain

$$\bar{k^{\left[ 2 \right]}}\left( t \right)=\frac{\sum_{j=1}^{t} \left( \mathrm{In} \frac{t}{t_{j}}+1 \right)^{2}}{2t}-1$$

$$=\frac{\sum_{j=1}^{t} \left( \mathrm{In} t-\mathrm{In} t_{j}+1 \right)^{2}}{2t}-1$$

$$=\frac{\left( \mathrm{In} t \right)^{2}\sum_{j=1}^{t} 1-2\mathrm{In} t\sum_{j=1}^{t} \mathrm{In} t_{j}+\sum_{j=1}^{t} \left( \mathrm{In} t_{j} \right)^{2}}{2t}-1$$

$$\approx\frac{\left( \mathrm{In} t \right)^{2}t-2\mathrm{In} t\int_{1}^{t} \mathrm{In} xdx+\int_{1}^{t} \left( \mathrm{In} x \right)^{2}dx}{2t}-1$$

$$=\frac{3}{2}-\frac{\mathrm{In} t}{t}-\frac{2}{t},\cdots(S7)$$

and, therefore,

$$\lim_{t\to\infty} \bar{k^{\left[ 2 \right]}}\left( t \right)=\frac{3}{2}.\cdots(S8)$$

In the same way, in the U_D_ model, since $k_{i}^{\left[ 1 \right]}(t)$ temporally develops in accordance with Eq. (18), the solution with $m=1$ is obtained as

$$k_{i}^{\left[ 1 \right]}\left( t \right)=\left( \frac{t}{t_{i}} \right)^{\frac{1}{2}}\cdots(S9)$$

under the initial condition (S5). Then, substituting Eq. (S9) into Eq. (S2), we obtain

$$\bar{k^{\left[ 2 \right]}}\left( t \right)=\frac{\sum_{j=1}^{t} \left\{ \left( \frac{t}{t_{j}} \right)^{\frac{1}{2}} \right\}^{2}}{2t}-1$$

$$=\frac{\sum_{j=1}^{t} \left( \frac{t}{t_{j}} \right)}{2t}-1$$

$$\approx\frac{t}{2t}\int_{1}^{t} \frac{dx}{x}-1$$

$$=\frac{1}{2}\mathrm{In} t-1$$

$$=\mathrm{In} \sqrt{t}-1$$

$$\approx\mathrm{In} \sqrt{t} \mathrm{for} t\gg1$$

$$\approx\mathrm{In}\sqrt{k^{\left[ 1 \right]}\left( t \right)}.\cdots\left( S10 \right)$$

**S2: An approximate relationship between** $\boldsymbol{k}_{\boldsymbol{i}}^{\left[ \boldsymbol{1} \right]}\boldsymbol{(t)}$ **and** $\boldsymbol{k}_{\boldsymbol{i}}^{\left[ \boldsymbol{2} \right]}\left( \boldsymbol{t} \right)$ **in the U_R_, U_D_, U_I_ models**

In the U_R_ model, $k_{i}^{\left[ 2 \right]}\left( t \right)$ develops temporally according to

$$\frac{d}{dt}k_{i}^{\left[ 2 \right]}\left( t \right)=\frac{k_{i}^{\left[ 1 \right]}\left( t \right)}{t+m_{int}-1}.\cdots(S11)$$

Dividing Eq. (S11) by Eq. (S3) on both sides of the equation to remove the denominator, we obtain

$$\frac{dk_{i}^{\left[ 2 \right]}\left( t \right)}{dk_{i}^{\left[ 1 \right]}(t)}=k_{i}^{\left[ 1 \right]}\left( t \right).\cdots(S12)$$

Since $k_{i}^{\left[ 1 \right]}(t)$ and $k_{i}^{\left[ 2 \right]}\left( t \right)$ are monotone increasing functions in the range of $t_{i}$ and $t$, respectively, Eq. (S12) can be integrated using the Stieltjes integration under the initial condition (S5) as

$$\int_{t_{i}}^{t} dk_{i}^{\left[ 2 \right]}\left( s \right)=\int_{t_{i}}^{t} k_{i}^{\left[ 1 \right]}\left( t \right)dk_{i}^{\left[ 1 \right]}\left( s \right)$$

$$\to k_{i}^{\left[ 2 \right]}\left( t \right)-k_{i}^{\left[ 2 \right]}\left( t_{i} \right)=\frac{1}{2}\left( k_{i}^{\left[ 1 \right]}\left( t \right) \right)^{2}-\frac{1}{2}\left( k_{i}^{\left[ 1 \right]}\left( t_{i} \right) \right)^{2}$$

$$\to k_{i}^{\left[ 2 \right]}\left( t \right)=\frac{1}{2}\left( k_{i}^{\left[ 1 \right]}\left( t \right) \right)^{2}+C_{i} (C_{i}\equiv k_{i}^{\left[ 2 \right]}\left( t_{i} \right)-\frac{1}{2}m^{2}).\cdots(S13)$$

Therefore, the relationship between $k_{i}^{\left[ 1 \right]}(t)$ and $k_{i}^{\left[ 2 \right]}\left( t \right)$ in the U_R_ model is

$$k_{i}^{\left[ 2 \right]}\left( t \right)=\frac{1}{2}\left( k_{i}^{\left[ 1 \right]}\left( t \right) \right)^{2} \mathrm{for} t\gg t_{i}.\cdots(S14)$$

Thus, $k_{i}^{\left[ 2 \right]}(t)$ is proportional to $k_{i}^{\left[ 1 \right]}(t)$ to a scaling exponent of 2. However, since, in the U_R_ model, a hub $i$ with a remarkably high value of $k_{i}^{\left[ 1 \right]}(t)$ does not occur, $k_{i}^{\left[ 2 \right]}(t)$ and $k^{\left[ 2 \right]}(t)$ do not increase significantly.

In the U_D_ model, dividing Eq. (19) by Eq. (18) on both sides of the equation to remove the denominator, we obtain

$$\frac{dk_{i}^{\left[ 2 \right]}\left( t \right)}{dk_{i}^{\left[ 1 \right]}(t)}=\frac{k_{i}^{\left[ 1 \right]}\left( t \right)+k_{i}^{\left[ 2 \right]}\left( t \right)}{k_{i}^{\left[ 1 \right]}\left( t \right)}.\cdots(S15)$$

Using the variable transformation, $s_{i}^{\left[ 2 \right]}\left( t \right)={k_{i}^{\left[ 2 \right]}\left( t \right)}/{k_{i}^{\left[ 1 \right]}\left( t \right)}$, Eq. (S15) is rewritten as

$$\frac{ds_{i}^{\left[ 2 \right]}\left( t \right)}{dk_{i}^{\left[ 1 \right]}(t)}=\frac{1}{k_{i}^{\left[ 1 \right]}\left( t \right)}.\cdots(S16)$$

Since $k_{i}^{\left[ 1 \right]}(t)$ and $s_{i}^{\left[ 2 \right]}\left( t \right)$ are monotone increasing functions in the range of $t_{i}$ and $t$, respectively, Eq. (S16) can be integrated using the Stieltjes integration under the initial condition (S5) as

$$\int_{t_{i}}^{t} ds_{i}^{\left[ 2 \right]}\left( s \right)=\int_{t_{i}}^{t} \frac{1}{k_{i}^{\left[ 1 \right]}\left( s \right)}dk_{i}^{\left[ 1 \right]}\left( s \right)$$

$$\to s_{i}^{\left[ 2 \right]}\left( t \right)-s_{i}^{\left[ 2 \right]}\left( t_{i} \right)=\log k_{i}^{\left[ 1 \right]}\left( t \right)-\log k_{i}^{\left[ 1 \right]}\left( t_{i} \right)$$

$$\to\frac{k_{i}^{\left[ 2 \right]}\left( t \right)}{k_{i}^{\left[ 1 \right]}\left( t \right)}-\frac{k_{i}^{\left[ 2 \right]}\left( t_{i} \right)}{k_{i}^{\left[ 1 \right]}\left( t_{i} \right)}=\log k_{i}^{\left[ 1 \right]}\left( t \right)-\log k_{i}^{\left[ 1 \right]}\left( t_{i} \right).\cdots(S17)$$

Therefore, the relationship between $k_{i}^{\left[ 1 \right]}(t)$ and $k_{i}^{\left[ 2 \right]}\left( t \right)$ in the U_D_ model is

$$k_{i}^{\left[ 2 \right]}\left( t \right)=k_{i}^{\left[ 1 \right]}\left( t \right)\log k_{i}^{\left[ 1 \right]}\left( t \right)+C_{i}k_{i}^{\left[ 1 \right]}\left( t \right) (C_{i}\equiv\frac{k_{i}^{\left[ 2 \right]}\left( t_{i} \right)}{m}-\log m).\cdots(S18)$$

In the U_I_ model, dividing Eq. (22) by Eq. (21) on both sides of the equation to remove the denominator, we obtain

$$\frac{dk_{i}^{\left[ 2 \right]}\left( t \right)}{dk_{i}^{\left[ 1 \right]}(t)}=\frac{k_{i}^{\left[ 1 \right]}\left( t \right)\left( k_{i}^{\left[ 1 \right]}\left( t \right)-1 \right)+k_{i}^{\left[ 3 \right]}(t)}{k_{i}^{\left[ 2 \right]}\left( t \right)}.\cdots(S19)$$

Since $k_{i}^{\left[ 1 \right]}(t)$ and $k_{i}^{\left[ 2 \right]}\left( t \right)$ are monotone increasing functions of $t$ in the range of $t_{i}$ and $t$, respectively, Eq. (S19) can be integrated using the Stieltjes integration under the initial condition (S5) as

$$\int_{t_{i}}^{t} k_{i}^{\left[ 2 \right]}\left( s \right)dk_{i}^{\left[ 2 \right]}\left( s \right)=\int_{t_{i}}^{t} \left\{ k_{i}^{\left[ 1 \right]}\left( s \right)\left( k_{i}^{\left[ 1 \right]}\left( s \right)-1 \right)+k_{i}^{\left[ 3 \right]}(s) \right\}dk_{i}^{\left[ 1 \right]}(s)$$

$$\to\frac{1}{2}\left( k_{i}^{\left[ 2 \right]}\left( t \right) \right)^{2}-\frac{1}{2}\left( k_{i}^{\left[ 2 \right]}\left( t_{i} \right) \right)^{2}$$

$$=\frac{1}{3}\left( k_{i}^{\left[ 1 \right]}\left( t \right) \right)^{3}-\frac{1}{3}\left( k_{i}^{\left[ 1 \right]}\left( t_{i} \right) \right)^{3}-\frac{1}{2}\left( k_{i}^{\left[ 1 \right]}\left( t \right) \right)^{2}+\frac{1}{2}\left( k_{i}^{\left[ 1 \right]}\left( t_{i} \right) \right)^{2}+\int_{t_{i}}^{t} k_{i}^{\left[ 3 \right]}\left( s \right)dk_{i}^{\left[ 1 \right]}\left( s \right).\cdots(S20)$$

Therefore, the relationship between $k_{i}^{\left[ 1 \right]}(t)$ and $k_{i}^{\left[ 2 \right]}\left( t \right)$ in the U_R_ model is

$$\frac{1}{2}\left( k_{i}^{\left[ 2 \right]}\left( t \right) \right)^{2}=\frac{1}{3}\left( k_{i}^{\left[ 1 \right]}\left( t \right) \right)^{3}-\frac{1}{2}\left( k_{i}^{\left[ 1 \right]}\left( t \right) \right)^{2}+\int_{t_{i}}^{t} k_{i}^{\left[ 3 \right]}\left( s \right)dk_{i}^{\left[ 1 \right]}\left( s \right)+C_{i}$$

$$\left( C_{i}\equiv\frac{1}{2}\left( k_{i}^{\left[ 2 \right]}\left( t_{i} \right) \right)^{2}-\frac{1}{3}m^{3}+\frac{1}{2}m^{2} \right).\cdots\left( S21 \right)$$

Here, if it can be assumed that

$$k_{i}^{\left[ 2 \right]}\left( t \right)=c_{i}^{\left[ 2 \right]}\left( k_{i}^{\left[ 1 \right]}\left( t \right) \right)^{\alpha},\cdots(S22)$$

$$k_{i}^{\left[ 3 \right]}\left( t \right)=c_{i}^{\left[ 3 \right]}\left( k_{i}^{\left[ 1 \right]}\left( t \right) \right)^{\beta},\cdots(S23)$$

substituting Eqs. (S22) and (S23) into Eq. (S21), we obtain

$$\frac{\left( c_{i}^{\left[ 2 \right]} \right)^{2}}{2}\left( k_{i}^{\left[ 1 \right]}\left( t \right) \right)^{2\alpha}=\frac{1}{3}\left( k_{i}^{\left[ 1 \right]}\left( t \right) \right)^{3}-\frac{1}{2}\left( k_{i}^{\left[ 1 \right]}\left( t \right) \right)^{2}+\frac{c_{i}^{\left[ 3 \right]}}{\beta+1}\left( k_{i}^{\left[ 1 \right]}\left( t \right) \right)^{\beta+1}+C_{i}^{'}$$

$$\left( C_{i}^{'}\equiv\frac{1}{2}\left( k_{i}^{\left[ 2 \right]}\left( t_{i} \right) \right)^{2}-\frac{c_{i}^{\left[ 3 \right]}}{\beta+1}m^{\beta+1}-\frac{1}{3}m^{3}+\frac{1}{2}m^{2} \right).\cdots\left( S24 \right)$$

As a rough estimate, we can determine from Eq. (S24) that, if the condition $3\geq\beta+1$, that is,

$$\beta\leq2\cdots(S25)$$

is satisfied, the first term is the leading term compared with the third term on the right side. Then, the consistency of Eq. (S24) is established under the relationship between $\alpha$ and $\beta$, $2\alpha=3$, that is,

$$\alpha=\frac{3}{2}.\cdots(S26)$$

Thus, under condition (S26), $k_{i}^{\left[ 2 \right]}\left( t \right)$ grows roughly according to $k_{i}^{\left[ 1 \right]}\left( t \right)$ to a scaling exponent of $1.5$, that is, $k_{i}^{\left[ 2 \right]}\left( t \right)\propto{(k_{i}^{\left[ 1 \right]}\left( t \right))}^{1.5}$. At this time, the rapid increase in $k_{i}^{\left[ 2 \right]}\left( t \right)$ spreads a converged region centring on a node $i$, and the growth of the region propagates from the centre to the outer side. This type of growth can be termed “active growth” because it leads the growth within the region.

On the contrary, if

$$\beta>2\cdots(S27)$$

is fulfilled, the third term is the leading term, and the consistency of Eq. (S24) is established under the relationship between $\alpha$ and $\beta$, $2\alpha=\beta+1$, that is,

$$\alpha=\frac{\beta+1}{2}.\cdots(S28)$$

Under condition (S27), the growth rate of $k_{i}^{\left[ 2 \right]}\left( t \right)$ is dominated by the leading term of $k_{i}^{\left[ 3 \right]}\left( t \right)$. Therefore, a growth pattern such that $k_{i}^{\left[ 3 \right]}\left( t \right)$ attracts $k_{i}^{\left[ 2 \right]}\left( t \right)$ and $k_{i}^{\left[ 2 \right]}\left( t \right)$ attracts $k_{i}^{\left[ 1 \right]}\left( t \right)$ appears. This pattern can be described as “passive growth” because it reads the growth outside the region.

**S3: Derivation of the utility-based preferential attachment** **model with** $\boldsymbol{n=2}$ **and an approximate relationship between** $\boldsymbol{k}_{\boldsymbol{i}}^{\left[ \boldsymbol{1} \right]}\boldsymbol{(t)}$ **and** $\boldsymbol{k}_{\boldsymbol{i}}^{\left[ \boldsymbol{2} \right]}\left( \boldsymbol{t} \right)$ **in this model**

Substituting Eq. (14) into Eq. (10), we obtain Eq. (15), that is,

$$\frac{d}{dt}k_{i}^{\left[ 1 \right]}\left( t \right)=m\frac{g\left( c,b_{1} \right)k_{i}^{\left[ 1 \right]}\left( t \right)+b_{2}k_{i}^{\left[ 2 \right]}\left( t \right)}{\sum_{j=1}^{t} \left\{ g\left( c,b_{1} \right)k_{i}^{\left[ 1 \right]}\left( t \right)+b_{2}k_{i}^{\left[ 2 \right]}\left( t \right) \right\}}.\cdots(15)$$

Notably here,

$$k_{i}^{\left[ 2 \right]}\left( t \right)=\sum_{\alpha=1} k_{i_{\alpha}}^{\left[ 1 \right]}(t),\cdots(S29)$$

where $k_{i_{\alpha}}^{\left[ 1 \right]}(t)$ is the number of the direct links of node $i_{\alpha}$, an existing node directly connected to node $i$ (see Fig. 1). Applying Eq. (20) to each term on the right side of Eq. (S29), we obtain

$$\frac{d}{dt}k_{i_{\alpha}}^{\left[ 1 \right]}\left( t \right)=m\frac{g\left( c,b_{1} \right)k_{i_{\alpha}}^{\left[ 1 \right]}\left( t \right)+b_{2}k_{i_{\alpha}}^{\left[ 2 \right]}\left( t \right)}{\sum_{j=1}^{t} \left\{ g\left( c,b_{1} \right)k_{j}^{\left[ 1 \right]}\left( t \right)+b_{2}k_{j}^{\left[ 2 \right]}\left( t \right) \right\}}.\cdots(S30)$$

Further, since the relationship

$$k_{i_{\alpha}}^{\left[ 2 \right]}\left( t \right)=\left( k_{i}^{\left[ 1 \right]}\left( t \right)-1 \right)+\sum_{\beta=1} k_{i_{\alpha(\beta)}}^{\left[ 3 \right]}(t),\cdots(S31)$$

is established (see Fig. 1), substituting Eq. (S31) into Eq. (S30), we obtain

$$\frac{d}{dt}k_{i_{\alpha}}^{\left[ 1 \right]}\left( t \right)=m\frac{g\left( c,b_{1} \right)k_{i_{\alpha}}^{\left[ 1 \right]}\left( t \right)+b_{2}\left\{ \left( k_{i}^{\left[ 1 \right]}\left( t \right)-1 \right)+\sum_{\beta=1} k_{i_{\alpha\left( \beta\right)}}^{\left[ 3 \right]}\left( t \right) \right\}}{\sum_{j=1}^{t} \left\{ g\left( c,b_{1} \right)k_{j}^{\left[ 1 \right]}\left( t \right)+b_{2}k_{j}^{\left[ 2 \right]}\left( t \right) \right\}}$$

$$=m\frac{g\left( c,b_{1} \right)k_{i_{\alpha}}^{\left[ 1 \right]}\left( t \right)+b_{2}\left( k_{i}^{\left[ 1 \right]}\left( t \right)-1 \right)+b_{2}\sum_{\beta=1} k_{i_{\alpha\left( \beta\right)}}^{\left[ 3 \right]}\left( t \right)}{\sum_{j=1}^{t} \left\{ g\left( c,b_{1} \right)k_{j}^{\left[ 1 \right]}\left( t \right)+b_{2}k_{j}^{\left[ 2 \right]}\left( t \right) \right\}}.\cdots(S32)$$

Further, taking the sum of$\alpha$ at both sides of Eq. (S32), we obtain

$$\sum_{\alpha=1}^{k_{i}^{[1]}} \left( \frac{d}{dt}k_{i_{\alpha}}^{\left[ 1 \right]}\left( t \right) \right)$$

$$=m\frac{g\left( c,b_{1} \right)\sum_{\alpha=1}^{k_{i}^{\left[ 1 \right]}(t)} k_{i_{\alpha}}^{\left[ 1 \right]}\left( t \right)+b_{2}\sum_{\alpha=1}^{k_{i}^{\left[ 1 \right]}(t)} \left( k_{i}^{\left[ 1 \right]}\left( t \right)-1 \right)+b_{2}\sum_{\alpha=1}^{k_{i}^{\left[ 1 \right]}(t)} \sum_{\beta=1} k_{i_{\alpha\left( \beta\right)}}^{\left[ 3 \right]}\left( t \right)}{\sum_{j=1}^{t} \left\{ g\left( c,b_{1} \right)k_{j}^{\left[ 1 \right]}\left( t \right)+b_{2}k_{j}^{\left[ 2 \right]}\left( t \right) \right\}}.\cdots(S33)$$

Next, using Eq. ($S29$) and the relationships

$$\sum_{\alpha=1}^{k_{i}^{[1]}} 1=k_{i}^{\left[ 1 \right]}\left( t \right),\cdots(S34)$$

$$\sum_{\alpha=1}^{k_{i}^{\left[ 1 \right]}(t)} \sum_{\beta=1} k_{i_{\alpha\left( \beta\right)}}^{\left[ 3 \right]}\left( t \right)=k_{i}^{\left[ 3 \right]}(t),\cdots(S35)$$

we rewrite Eq. (S33) as

$$\frac{d}{dt}k_{i}^{\left[ 2 \right]}\left( t \right)=m\frac{b_{2}\left( k_{i}^{\left[ 1 \right]}\left( t \right) \right)^{2}+\left\{ g\left( c,b_{1} \right)-b_{2} \right\}k_{i}^{\left[ 1 \right]}(t)+g\left( c,b_{1} \right)k_{i}^{\left[ 2 \right]}\left( t \right)+b_{2}k_{i}^{[3]}(t)}{\sum_{j=1}^{t} \left\{ g\left( c,b_{1} \right)k_{j}^{\left[ 1 \right]}\left( t \right)+b_{2}k_{j}^{\left[ 2 \right]}\left( t \right) \right\}}.\cdots(S36)$$

Thus, we obtain Eqs. (15) and (16) as

$$\frac{d}{dt}k_{i}^{\left[ 1 \right]}\left( t \right)=m\frac{g\left( c,b_{1} \right)k_{i}^{\left[ 1 \right]}\left( t \right)+b_{2}k_{i}^{\left[ 2 \right]}\left( t \right)}{\sum_{j=1}^{t} \left\{ g\left( c,b_{1} \right)k_{j}^{\left[ 1 \right]}\left( t \right)+b_{2}k_{j}^{\left[ 2 \right]}\left( t \right) \right\}},\cdots(15)$$

$$\frac{d}{dt}k_{i}^{\left[ 2 \right]}\left( t \right)=m\frac{b_{2}\left( k_{i}^{\left[ 1 \right]}\left( t \right) \right)^{2}+\left\{ g\left( c,b_{1} \right)-b_{2} \right\}k_{i}^{\left[ 1 \right]}(t)+g\left( c,b_{1} \right)k_{i}^{\left[ 2 \right]}\left( t \right)+b_{2}k_{i}^{[3]}(t)}{\sum_{j=1}^{t} \left\{ g\left( c,b_{1} \right)k_{j}^{\left[ 1 \right]}\left( t \right)+b_{2}k_{j}^{\left[ 2 \right]}\left( t \right) \right\}}.\cdots(16)$$

Dividing Eq. (16) by Eq. (15) on both sides of the equation to remove the denominator, we obtain

$$\frac{dk_{i}^{\left[ 2 \right]}\left( t \right)}{dk_{i}^{\left[ 1 \right]}\left( t \right)}=\frac{b_{2}\left( k_{i}^{\left[ 1 \right]}\left( t \right) \right)^{2}+\left\{ g\left( c,b_{1} \right)-b_{2} \right\}k_{i}^{\left[ 1 \right]}(t)+g\left( c,b_{1} \right)k_{i}^{\left[ 2 \right]}\left( t \right)+b_{2}k_{i}^{[3]}(t)}{g\left( c,b_{1} \right)k_{i}^{\left[ 1 \right]}\left( t \right)+b_{2}k_{i}^{\left[ 2 \right]}\left( t \right)}.\cdots(S37)$$

Since $k_{i}^{\left[ 1 \right]}(t)$ and $k_{i}^{\left[ 2 \right]}\left( t \right)$ are monotone increasing functions of $t$ in the range of $t_{i}$ and $t$, respectively, Eq. (S37) can be integrated using the Stieltjes integration under the initial condition (S5) as

$$g\left( c,b_{1} \right)\int_{t_{i}}^{t} k_{i}^{\left[ 1 \right]}\left( s \right)dk_{i}^{\left[ 2 \right]}\left( s \right)+b_{2}\int_{t_{i}}^{t} k_{i}^{\left[ 2 \right]}\left( s \right)dk_{i}^{\left[ 2 \right]}\left( s \right)=b_{2}\int_{t_{i}}^{t} \left( k_{i}^{\left[ 1 \right]}\left( s \right) \right)^{2}dk_{i}^{\left[ 1 \right]}\left( s \right)+\left\{ g\left( c,b_{1} \right)-b_{2} \right\}\int_{t_{i}}^{t} k_{i}^{\left[ 1 \right]}\left( s \right)dk_{i}^{\left[ 1 \right]}\left( s \right)+g\left( c,b_{1} \right)\int_{t_{i}}^{t} k_{i}^{\left[ 2 \right]}\left( s \right)dk_{i}^{\left[ 1 \right]}\left( s \right)+b_{2}\int_{t_{i}}^{t} k_{i}^{[3]}(s)dk_{i}^{\left[ 1 \right]}\left( s \right)$$

$$\to g\left( c,b_{1} \right)\int_{t_{i}}^{t} k_{i}^{\left[ 1 \right]}\left( s \right)\frac{dk_{i}^{\left[ 2 \right]}\left( s \right)}{dk_{i}^{\left[ 1 \right]}\left( s \right)}dk_{i}^{\left[ 1 \right]}\left( s \right)+b_{2}\left\{ \frac{1}{2}\left( k_{i}^{\left[ 2 \right]}\left( t \right) \right)^{2}-\frac{1}{2}\left( k_{i}^{\left[ 2 \right]}\left( t_{i} \right) \right)^{2} \right\}=b_{2}\left\{ \frac{1}{3}\left( k_{i}^{\left[ 1 \right]}\left( t \right) \right)^{3}-\frac{1}{3}\left( k_{i}^{\left[ 1 \right]}\left( t_{i} \right) \right)^{3} \right\}+\left\{ g\left( c,b_{1} \right)-b_{2} \right\}\left\{ \frac{1}{2}\left( k_{i}^{\left[ 1 \right]}\left( t \right) \right)^{2}-\frac{1}{2}\left( k_{i}^{\left[ 1 \right]}\left( t_{i} \right) \right)^{2} \right\}+g\left( c,b_{1} \right)\int_{t_{i}}^{t} k_{i}^{\left[ 2 \right]}\left( s \right)dk_{i}^{\left[ 1 \right]}\left( s \right)+b_{2}\int_{t_{i}}^{t} k_{i}^{\left[ 3 \right]}\left( s \right)dk_{i}^{\left[ 1 \right]}\left( s \right).\cdots(S38)$$

Here, if Eqs. (S22) and (S23) can be assumed, substituting Eqs. (S22) and (S23) into Eq. (S38), we obtain

$$\to g\left( c,b_{1} \right)\int_{t_{i}}^{t} k_{i}^{\left[ 1 \right]}\left( s \right)c_{i}^{\left[ 2 \right]}\left( k_{i}^{\left[ 1 \right]}\left( s \right) \right)^{\alpha-1}dk_{i}^{\left[ 1 \right]}\left( s \right)+b_{2}\left\{ \frac{1}{2}\left( c_{i}^{\left[ 2 \right]} \right)^{2}\left( k_{i}^{\left[ 1 \right]}\left( t \right) \right)^{2\alpha}-\frac{1}{2}\left( k_{i}^{\left[ 2 \right]}\left( t_{i} \right) \right)^{2} \right\}=b_{2}\left\{ \frac{1}{3}\left( k_{i}^{\left[ 1 \right]}\left( t \right) \right)^{3}-\frac{1}{3}\left( k_{i}^{\left[ 1 \right]}\left( t_{i} \right) \right)^{3} \right\}+\left\{ g\left( c,b_{1} \right)-b_{2} \right\}\left\{ \frac{1}{2}\left( k_{i}^{\left[ 1 \right]}\left( t \right) \right)^{2}-\frac{1}{2}\left( k_{i}^{\left[ 1 \right]}\left( t_{i} \right) \right)^{2} \right\}+g\left( c,b_{1} \right)c_{i}^{\left[ 2 \right]}\int_{t_{i}}^{t} \left( k_{i}^{\left[ 1 \right]}\left( s \right) \right)^{\alpha}dk_{i}^{\left[ 1 \right]}\left( s \right)+b_{2}c_{i}^{\left[ 3 \right]}\int_{t_{i}}^{t} \left( k_{i}^{\left[ 1 \right]}\left( s \right) \right)^{\beta}dk_{i}^{\left[ 1 \right]}\left( s \right)$$

$$\to g\left( c,b_{1} \right)c_{i}^{\left[ 2 \right]}\int_{t_{i}}^{t} \left( k_{i}^{\left[ 1 \right]}\left( s \right) \right)^{\alpha}dk_{i}^{\left[ 1 \right]}\left( s \right)+b_{2}\left\{ \frac{1}{2}\left( c_{i}^{\left[ 2 \right]} \right)^{2}\left( k_{i}^{\left[ 1 \right]}\left( t \right) \right)^{2\alpha}-\frac{1}{2}\left( k_{i}^{\left[ 2 \right]}\left( t_{i} \right) \right)^{2} \right\}=b_{2}\left\{ \frac{1}{3}\left( k_{i}^{\left[ 1 \right]}\left( t \right) \right)^{3}-\frac{1}{3}\left( k_{i}^{\left[ 1 \right]}\left( t_{i} \right) \right)^{3} \right\}+\left\{ g\left( c,b_{1} \right)-b_{2} \right\}\left\{ \frac{1}{2}\left( k_{i}^{\left[ 1 \right]}\left( t \right) \right)^{2}-\frac{1}{2}\left( k_{i}^{\left[ 1 \right]}\left( t_{i} \right) \right)^{2} \right\}+g\left( c,b_{1} \right)\int_{t_{i}}^{t} \left( k_{i}^{\left[ 1 \right]}\left( s \right) \right)^{\alpha}dk_{i}^{\left[ 1 \right]}\left( s \right)+b_{2}\frac{c_{i}^{\left[ 3 \right]}}{1+\beta}\left\{ \left( k_{i}^{\left[ 1 \right]}\left( t \right) \right)^{\beta+1}-\left( k_{i}^{\left[ 1 \right]}\left( t_{i} \right) \right)^{\beta+1} \right\}$$

$$\to b_{2}\left\{ \frac{1}{2}\left( c_{i}^{\left[ 2 \right]} \right)^{2}\left( k_{i}^{\left[ 1 \right]}\left( t \right) \right)^{2\alpha}-\frac{1}{2}\left( k_{i}^{\left[ 2 \right]}\left( t_{i} \right) \right)^{2} \right\}=b_{2}\left\{ \frac{1}{3}\left( k_{i}^{\left[ 1 \right]}\left( t \right) \right)^{3}-\frac{1}{3}m^{3} \right\}+\left\{ g\left( c,b_{1} \right)-b_{2} \right\}\left\{ \frac{1}{2}\left( k_{i}^{\left[ 1 \right]}\left( t \right) \right)^{2}-\frac{1}{2}m^{2} \right\}+b_{2}\frac{c_{i}^{\left[ 3 \right]}}{1+\beta}\left\{ \left( k_{i}^{\left[ 1 \right]}\left( t \right) \right)^{\beta+1}-m^{\beta+1} \right\}$$

$$\to\frac{b_{2}}{2}\left( c_{i}^{\left[ 2 \right]} \right)^{2}\left( k_{i}^{\left[ 1 \right]}\left( t \right) \right)^{2\alpha}=\frac{b_{2}}{3}\left( k_{i}^{\left[ 1 \right]}\left( t \right) \right)^{3}+\frac{g\left( c,b_{1} \right)-b_{2}}{2}\left( k_{i}^{\left[ 1 \right]}\left( t \right) \right)^{2}+\frac{b_{2}}{1+\beta}c_{i}^{\left[ 3 \right]}\left( k_{i}^{\left[ 1 \right]}\left( t \right) \right)^{\beta+1}+C_{i}$$

$$\left( C_{i}\equiv\frac{b_{2}}{2}\left( k_{i}^{\left[ 2 \right]}\left( t_{i} \right) \right)^{2}-\frac{b_{2}}{3}m^{3}-\frac{g\left( c,b_{1} \right)-b_{2}}{2}m^{2}-\frac{b_{2}}{1+\beta}c_{i}^{\left[ 3 \right]}m^{\beta+1} \right).\cdots(S39)$$

Here, we can roughly estimate the restriction of the powers $\alpha, \beta$ as in the case of Eq. (S24). That is, if the condition

$$\beta\leq2,\cdots(S25)$$

is fulfilled, $\alpha$ is estimated as

$$\alpha=\frac{3}{2}.\cdots(S26)$$

On the contrary, if the condition

$$\beta>2,\cdots(S27)$$

is fulfilled, $\alpha$ is estimated as

$$\alpha=\frac{\beta+1}{2}.\cdots(S28)$$

**S4: Mixed preferential attachment model**

In the Results section, we describe our investigation of the structural properties of the networks formed by the U_R_, U_D_, U_I_, U_M+_, and U_M-_ models, into which all nodes enter with the same attachment rule. However, the actual rules for attachment into a social network would vary from node to node. Therefore, as a more realistic analysis, we describe here our investigation of the structural properties of evolving networks into which each node enters by selecting one of the attachment rules of the U_R_, U_D_, and U_I_ models with equal probability.

Fig. S1a shows a network evolved through 50,000 steps by sequentially entering nodes based on the attachment rules of the U_R_, U_D_, and U_I_ models. This figure is created using the force-directed layout (ForceAtlas2) of the Gephi network visualization software. In this figure, boxes T_R_, T_D_, and T_I_ show a part of the network formed by the nodes (blue, orange, and green nodes) entered by the attachment rules of the U_R_, U_D_, and U_I_ models, respectively. Each box shows the nodes located around the local hubs and the links between the hubs. In particular, the number of links between the nodes in the box T_I_ is large because the effect of the mutual growth mechanism according to the preferential attachment based on indirect utility propagates growth around the hubs. So, the nodes in box T_I_ are concentrated around the local hubs. These structural properties also influence the growth of the nodes entered by each attachment rule.

Figs. S1b and S1c show the numerical simulation result of tracing the average of $k^{\left[ l \right]}(l=1,2)$ of the nodes in the networks evolved through 500,000 steps by sequentially entering the nodes with the distinct attachment rules of the U_R_, U_D_, and U_I_ models. Here, the error bar represents the standard deviation across 20 numerical simulations. The average of $k^{\left[ l \right]}(l=1,2)$ of the nodes entering by each attachment rule is defined mathematically as

$$E{(k^{\left[ l \right]}(t))}_{R}=\frac{1}{N_{R}\left( t \right)}\sum_{i\in N_{R}} k_{i}^{\left[ l \right]}(t),\cdots(S40)$$

$$E{(k^{\left[ l \right]}(t))}_{D}=\frac{1}{N_{D}\left( t \right)}\sum_{i\in N_{D}} k_{i}^{\left[ l \right]}(t),\cdots(S41)$$

$$E{(k^{\left[ l \right]}(t))}_{I}=\frac{1}{N_{I}\left( t \right)}\sum_{i\in N_{I}} k_{i}^{\left[ l \right]}(t),\cdots(S42)$$

where $N_{R}$(t), $N_{D}(t)$, and $N_{I}(t)$ are the number of nodes entering by each attachment rule at time $t$ and fulfill the constraint condition

$$N_{R}\left( t \right)+N_{D}\left( t \right)+N_{I}\left( t \right)=t.\cdots(S43)$$

Regarding $k^{\left[ 1 \right]}(t)$, the equation

$$E\left( k^{\left[ 1 \right]}\left( t \right) \right)_{Total}=\frac{1}{N_{R}\left( t \right)+N_{D}\left( t \right)+N_{I}\left( t \right)}\sum_{i\in N_{R}+N_{D}+N_{I}} k_{i}^{\left[ 1 \right]}\left( t \right)\approx\frac{2t}{t}=2\cdots(S44)$$

holds. As Fig. S1b indicates, only the nodes entered by the preferential attachment rule of the U_I_ continuously increase the value of $E{(k^{\left[ 1 \right]}(t))}_{I}$. Further, Fig. S1c indicates that the value of $E{(k^{\left[ 2 \right]}(t))}_{I}$ exceeds that of $E{(k^{\left[ l \right]}(t))}_{R}$ and $E{(k^{\left[ l \right]}(t))}_{D}$ and the preferential attachment rule of the U_I_ model forms the structure easily to obtain utility directly or indirectly. These results suggest that, among the preferential attachment rules presented, that of the U_I_ model prevails within the network.

Fig. S1d shows the patterns of direct growth and indirect growth of total nodes and nodes of each type, respectively. Although grown by nodes with three different preferences, the growth of all nodes shows a positive correlation between ${\Delta k}_{i}^{\left[ 1 \right]}(t)$and $\Delta k_{i}^{\left[ 2 \right]}(t)$ similar to growth by the indirect utility (Fig. 3b). This growth pattern also appears in the nodes of T_R_ and T_D_, because the mutual growth by the influx of T_I_ is applied to all existing nodes.


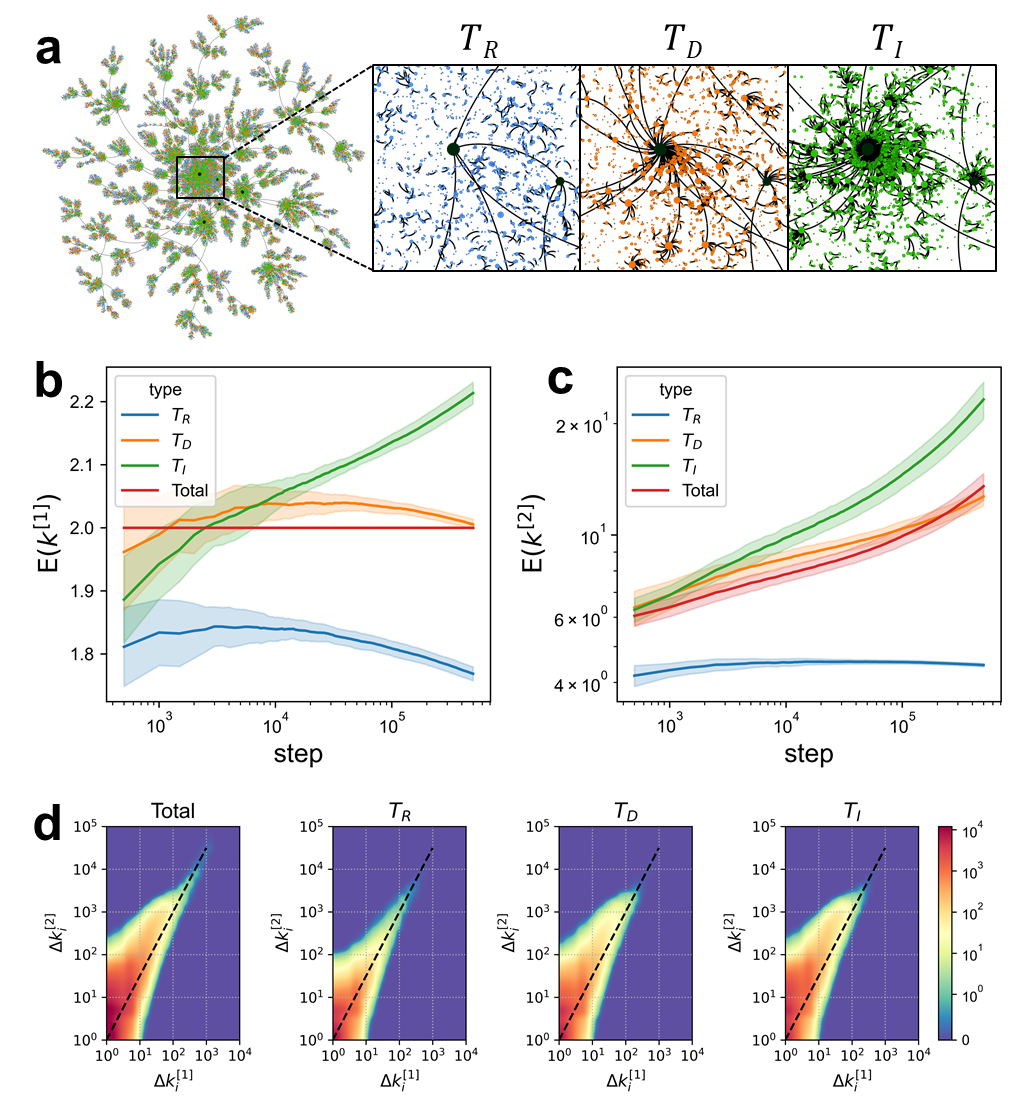


**Figure S1: Structural properties of a network evolved by alternately entering based on the attachment rules of the U_R_, U_D_, and U_I_ models.** Fig. S1a shows a network evolved through 50,000 steps by sequentially entered nodes based on the attachment rules of the U_R_, U_D_, and U_I_ models. Boxes T_R_, T_D_, and T_I_ show a part of the network formed by the nodes (blue, orange, and green nodes) entered based on the attachment rule of the U_R_ U_D_, and U_I_ models, respectively. The number of links between the nodes in box T_I_ is large. Figs. S1b and S1c show the numerical simulation result of tracing the average of $k^{\left[ l \right]}(l=1,2)$ of the nodes in networks evolved through 500,000 steps by sequentially entering nodes with the specific attachment rules of the U_R_, U_D_, and U_I_ models. Fig. S1b indicates that only the nodes entering based on the preferential attachment rule of the U_I_ model continuously increase $k^{\left[ 1 \right]}$ on average. Fig. S1c indicates that the average of $k_{i}^{\left[ 2 \right]}$ of the nodes entered based on the preferential attachment rule of the U_I_ model exceeds that of the other types of nodes and the preferential attachment rule of the U_I_ model forms the structure easily to obtain utility directly or indirectly. These results suggest that, among the preferential attachment rules presented, that of the U_I_ model prevails in the network. Fig. S1d show a set of heat maps between $\Delta k_{i}^{\left[ 1 \right]}(t)$ and $\Delta k_{i}^{\left[ 2 \right]}(t)$ of total nodes and nodes of each type, respectively. The dashed lines are theoretical expectation of mutual growth by preferential attachment base on indirect utility (see supplementary Information S2). Areas with no growth in direct and indirect growth are excluded for convenience on log scale.
